# Wnt11 Is Required for Oriented Migration of Dermogenic Progenitor Cells from the Dorsomedial Lip of the Avian Dermomyotome

**DOI:** 10.1371/journal.pone.0092679

**Published:** 2014-03-26

**Authors:** Gabriela Morosan-Puopolo, Ajeesh Balakrishnan-Renuka, Faisal Yusuf, Jingchen Chen, Fangping Dai, Georg Zoidl, Timo H.-W. Lüdtke, Andreas Kispert, Carsten Theiss, Mohammed Abdelsabour-Khalaf, Beate Brand-Saberi

**Affiliations:** 1 Department of Anatomy and Molecular Embryology, Ruhr-University of Bochum, Bochum, Germany; 2 Department of Molecular Embryology, Freiburg University, Freiburg, Germany; 3 Faculty of Biology, Freiburg University, Freiburg, Germany; 4 Institute for Molecular Biology, Medizinische Hochschule Hannover, Hannover, Germany; 5 Institute of Anatomy and Cell Biology, Department of Molecular Embryology, Albert-Ludwigs University, Freiburg, Germany; Heart Science Centre, Imperial College London, United Kingdom

## Abstract

The embryonic origin of the dermis in vertebrates can be traced back to the dermomyotome of the somites, the lateral plate mesoderm and the neural crest. The dermal precursors directly overlying the neural tube display a unique dense arrangement and are the first to induce skin appendage formation in vertebrate embryos. These dermal precursor cells have been shown to derive from the dorsomedial lip of the dermomyotome (DML). Based on its expression pattern in the DML, Wnt11 is a candidate regulator of dorsal dermis formation. Using EGFP-based cell labelling and time-lapse imaging, we show that the *Wnt11* expressing DML is the source of the dense dorsal dermis. Loss-of-function studies in chicken embryos show that *Wnt11* is indeed essential for the formation of dense dermis competent to support cutaneous appendage formation. Our findings show that dermogenic progenitors cannot leave the DML to form dense dorsal dermis following *Wnt11* silencing. No alterations were noticeable in the patterning or in the epithelial state of the dermomyotome including the DML. Furthermore, we show that *Wnt11* expression is regulated in a manner similar to the previously described early dermal marker *cDermo-1*. The analysis of *Wnt11* mutant mice exhibits an underdeveloped dorsal dermis and strongly supports our gene silencing data in chicken embryos. We conclude that Wnt11 is required for dense dermis and subsequent cutaneous appendage formation, by influencing the cell fate decision of the cells in the DML.

## Introduction

The presence of a connective tissue layer of the skin, called dermis, is the prerequisite for the development of cutaneous appendages. The somitic origin of the back dermis has been shown by Mauger in 1972 [1], [2] and later on, using quail-chick grafting technique, the medial origin of the dorsal dermis was demonstrated [3] During embryonic development, dermis in vertebrates takes its origin from three different sources. The dense dorsal dermis, which will be addressed mainly in this work, originates from the medial and central regions of the dermomyotome [4], the cranio-facial and cervical dermis is formed by neural crest cells [5], while the ventro-lateral trunk and the limb dermis are derived from the lateral plate mesoderm [1], [2]. In addition to being a source for the dorsal dermis, the dermomyotomes also give rise to striated muscle, blood and lymphatic vessels, and in the neck region to the scapular blade [6], [7], [8], [9], [10], [11].

The development of the dermal layer of the skin and its appendages is a complex and highly regulated process. Although late stages of dermis development and notably the formation of cutaneous appendages have been studied to some extent, molecular data on the early fate of dermal progenitor cells has only begun to surface [12], [13], [14], [15], [16]. The progenitor cells for the dermis of the back are established during somitic compartmentalization, while their differentiation into dermis as connective tissue layer of the skin occurs only later during development. In our study, we focus on the early specification of dermal progenitors and their oriented migration towards the dorsal midline which is shown here to be dependent on the presence of Wnt11. Wnt11 has previously been reported to maintain the DML in an epithelial state [17], however, our data suggest that EMT of dermogenic progenitors relies on Wnt11.

Based on the spatially restricted expression pattern of a number of regulatory genes in chicken embryos, the dermomyotome has been divided into a medial, a central and a lateral region. All three regions can be distinguished by markers, such as *Wnt11* in the DML, *En-1* in the central region and *Sim1* in the lateral region [14]. As well as being a source for the dermal precursor cells that collect under the ectoderm to form the dorsal dermis, the dermomyotome is furthermore a source of myoblasts that enter the myotome [18], [19], [20]. These regions of the dermomyotome are, thus, consisting of multipotent progenitor cells that yield myogenic and dermogenic precursors determined by the surrounding signals. While the DML and the central dermomyotome yield the dermal progenitors destined to form the dense dermis above the neural tube, the lateral part corresponding to the *Sim1* positive region is responsible for the loose dermis located in the normally apteric region of the chicken back [15].

In chicken embryos, the first visible signs of dermis development appear from day E5 (HH26) onwards, and in mouse, at an equivalent developmental stage day 9.5 (E9.5) [16], [21]. In the chicken embryo, the somite-derived dermal progenitor cells undergo EMT and organize to form a mesenchyme of noticeable thickness underneath the ectoderm between E3 and E5, adjacent to the neural tube and the differentiating myotome. It is only by E6 that the mesenchyme condenses locally and on E7 the epithelio-mesenchymal dialogue between dermis and epidermis is initiated which will eventually lead to the morphogenesis of cutaneous appendages.

The earliest dermis marker described to date is *Twist2* (*mDermo-1*) in the mouse [22] and its orthologue *cDermo-1* in the chicken [23]. The expression of *Twist2* appears first at E10.5 in cells of mesodermal and mesectodermal origin. Its expression in the dermal progenitor cells increases progressively and is prominently detectable under the overlying ectoderm from E12.5 onwards. The expression of *Twist2* (*mDermo-1*) in this domain of the prospective dermis is maintained until birth. In the chicken embryo, *cDermo-1* has been shown to influence the density of the dorsal dermis and to act upstream of *Fgf10* during dermis development [24]. Overexpression of *cDermo-1* leads to an increase in dense dermis with formation of ectopic and larger feather buds [24]. *cDermo-1* expression is controlled by BMP signaling and can be inhibited by the BMP antagonist Noggin [24], [25].

Although the origin of the dorsal dermis has been an object of interest for several research groups, so far no functional experimental data is available showing the direct relationship between gene expression in the dermomyotome and dermis formation. Based on its temporo-spatial expression pattern in the dorsomedial lip (DML) of the avian dermomyotome and subectodermal mesenchyme [26], *Wnt11* has been proposed to play a role during dermis development in addition to its described role in myogenesis [27]. A comparable expression pattern of *Wnt11* has been described in mice in the DML starting from E9.5 [28].

During somite development, two essential processes involving the cellular organization of the embryonic tissue occur: mesenchymal-epithelial transition (MET) occurs during somitogenesis and epithelio-mesenchymal transition (EMT) occurs during compartment formation and differentiation of the somites. *Snail1* is a member of the Snail family of zinc-finger transcription factors with an important role in triggering EMT during the development of the neural crest and somites [29]. So this marker is playing a key role in normal chicken embryo development and the cell fate decision of the cells located in the somites.

The present work aims to investigate the role of Wnt11 in dense dorsal dermis formation which is derived from the medial somite. Its function is analysed by means of loss-of-function experiments in the chicken and mouse embryo. We are able to show that knock-down of *Wnt11* in chicken embryos leads to compromised dense dorsal dermis formation due to a defective recruitment of dermal progenitor cells from the DML, causing a delay in feather bud development. This is corroborated by the analysis of knock-out mice for *Wnt11* which demonstrates a decrease in dorsal dermis thickness with a reduced number of hair follicles in comparison with their WT littermates. qPCR analysis for several skin markers in wild-type mice and homozygous *Wnt11* mutants points towards a defect in the formation of dense dorsal dermis in the absence of *Wnt11* as seen by a significant decrease in the number of *Twist2* (*mDermo-1*)-positive cells. In addition, we show that *Wnt11* is regulated in a fashion similar to *cDermo-1* in response to dermogenic and dermis inhibitory signals suggesting the involvement of Wnt11 and Dermo-1 in a common pathway controlling dermis formation.

## Materials and Methods

### Ethics Statement

According to German legislation, the use of embryonic vertebrates in an animal experiment needs approval only if the animal is in the last third of its embryonic development. In the case of chicken, this means that experiments done on animals before embryonic day 14 (E14) are not regarded as an animal experiment by the Tierschutzgesetz, and therefore, do not need approval or governmental permission.

The chicken embryos sacrificed for this work were between developmental stages HH21 (E3.5) and HH38 (E12). All embryos were sacrificed at the end of the study by opening the shell and tearing the allantois and amnion with forceps. Thereafter, the embryos were immersed in 4% PFA/PBS solution for fixation. No permits were required for the described study, which complied with all relevant regulations.

The mice embryos used in this study were provided by Medizinische Hochschule Hannover and were previously described [30]. All animal work conducted for this study was approved by H. Hedrich, state head of the animal facility at Medizinische Hochschule Hannover and performed according to German legislation. All mice were maintained on an outbred (NMRI) background. Mice were kept with regulated temperature embryos, heterozygous mice were intercrossed. Vaginal plugs were checked in the morning after mating, for timed pregnancies noon was taken as E0.5. Female mice were sacrificed by cervical dislocation. Embryos were harvested in PBS, decapitated, fixed in 4% paraformaldehyde overnight and stored in 100% methanol at –20°C before further use.

Genomic DNA prepared from yolk sacs or tail biopsies was used for genotyping by PCR.

### Chicken Embryos

Fertilized chicken eggs (White Leghorn, *Gallus domesticus*) were obtained from a local poultry farmer in Freiburg and a local breeder in Möhnesee. The eggs were incubated at 37°C under 80% humidity in a poultry egg incubator (Ehret). The progress of development was determined according to the staging system of [31].

### *In ovo* Electroporation (IOE)

Electroporation of the chicken embryos was performed as described before [32], [33]. Care was taken in the placement of the electrodes for effective transfection of the DML. Electroporation was carried out using the Intracel TSS20 OVODYNE Electroporator. Following electroporation, the eggs were sealed with tape and reincubated. The first EGFP expression could be detected 4 hours after electroporation.

### Gene Silencing Studies

The target sequences for *Wnt11* were selected using RNAi target selection tools from Ambion and Promega. Four target sites in the chicken *Wnt11* mRNA were chosen for the purpose of gene silencing (see Table S1). The selected nucleotide sequences chosen for targeting were subjected to nucleotide-nucleotide BLAST (blastn) analysis provided on the NCBI server (www.ncbi.nlm.nih.gov/BLAST) prior to the synthesis of corresponding shRNA-expressing constructs. This was done to precheck possible off-target silencing. For further preparation of the constructs two bi-cistronic vector systems were used. These vectors that had been modified from the *pCMV-EGFP* vector, differ in the type of the RNA polymerase III promoter, which is either H1 or U6. They share the *EGFP* sequence and the insertion place for the shRNA cistron [33]. The *pEGFP-H1-sh*RNA and *pEGFP*-*U6-sh*RNA vectors were pre-restricted with *BamHI* and *HindIII* enzymes. Each of the shRNA encoding cistrons was introduced into both mentioned vectors. The associated *EGFP* reporter sequence incorporated into the shRNA-expressing constructs permitted to detect the success and position of DML transfection. The efficiency of all four target sequences was individually tested by *in ovo* electroporation. Eventually for the silencing experiments, two cocktails were used one containing the *H1* and the other one containing the *U6* promoter.

Additional approaches employed for interfering with *Wnt11* expression or signaling, repectively, included a miRNA-RFP construct targeted against *Wnt11* (obtained from the lab of Christoph Marcelle, Monash University, Australia) [27] and a dominant-negative (DN) construct for *Wnt11* (from the lab of Philippa Francis-West, London) [34]. The DN-*Wnt11* RCAS was co-electroporated along with an EGFP reporter vector.

The silencing efficiency of *Wnt11* RNAi vectors was assessed by ISH analysis of embryos, transfected with an mRNA probe specific for avian *Wnt11*.

Controls were performed by electroporation of scrambled DNA with an EGFP reporter.

### *In Situ* Hybridization

Embryos, electroporated with *Wnt11* RNAi constructs, were tested using the following probes: *cDermo-1* probe of 1.029 kb [23]; *En-1* probe of 800 bp [35]; *Paraxis* probe of 1,2 kb [36]; *Pax3* probe of 660 bp [37]; *Shh* probe of 1,5 kb [38]; chicken *Wnt11* probe of 1000 bp [39]; *Sim1* 600 bp in pBlueSkript and *Snail1* 550 bp pGEMT Easy probes were kind gifts from Olivier Pourquié (IGBMC, Illkirch, France). *mTwist2* probe (400 bp insert in pT7T3) was kindly provided by Ernst-Martin Füchtbauer (Aarhus University, Denmark). The riboprobes were labeled using a digoxigenin RNA labeling kit from (Roche Diagnostics, Germany). Whole-mount *in situ* hybridization was performed as described previously [40].

### TUNEL Assay

The TUNEL (Terminal deoxynucleotidyl transferase dUTP nick end labeling) assay for detection of apoptotic cells was performed on paraffin sections. Tissue sections were treated with 0.5% pepsin in Aqua, HCl, at 37°C. For positive controls bovine pancreas DNase I treatment was performed for 30 minutes at 37°C.

All sections were quenched of endogenous peroxidase in 3% hydrogen peroxidase in methanol for 5 min.

For TdT labeling the sections were incubated for 10 min in TdT buffer and then incubated in labeling mixture for one hour at 37°C.

The sections were incubated with Streptavidin-HRP for 30 minutes at room temperature.

For AEC (3-amino-9-ethylcarbazole) reaction one AEC substrate reagent was prepared (4 ml deionized water, 2 drops Acetate Buffer, 1 drop AEC Chromogen, Sigma, 1 drop 3% hydrogen peroxide). Two drops of this substrate reagent were applied on each slide, for 10 minutes.

### Bead Implantation

Heparin-coated acrylic beads were used, approximately 40–80 μm in diameter (Sigma, Germany). Before being transferred into the BMP2 protein solution, the beads were rinsed 2–3 times for a few hours in PBS solution.

After the washing steps in PBS, the beads were further soaked in BMP2 protein solution (100 μg/μl). For obtaining different concentrations, the proteins were diluted in PBS+0.1% bovine serum albumin (BSA). BMP2 protein was obtained from the Genetics Institute, Cambridge, MA, USA.

### HE (Hematoxylin-Eosin) Staining

Paraffin sections of *Wnt11* mutant mice and wild-type mice were first deparaffinized. The sections were washed twice for 5 minutes in Rotihistol and then twice for 1 minute in 100% ethanol, followed by short washing steps of 30″–45″ in 95%, 90%, 80%, 70%, 50% and 30% ethanol. Finally, a short wash in ddH_2_O was followed by a 3–4 minutes wash in Hemalaun, and 10 minutes wash in running water. 1.5 minutes in 0.1% eosin in 70% ethanol were followed by short washes in 70%, 80%, 90%, 2×100% and 2× rotihistol. The slides were embedded in Entellan and then covered with cover slips.

### *Wnt11* Knock-out Mice and Dermis Volume

*Wnt11* knock-out mice were created by using a targeting construct containing 4.8 kb of 5′ and 4.0 kb of 3′ homology regions, which was transfected into 5×10 7 R1 ES cells derived from 129 Sv strain [41] using a BioRad pulser.

*Wnt11* homozygous mutant mice (W*nt11* ^−/−^) were identified by Southern blot and genomic PCR analyses. The absence of the exons IV and V in the targeted allele was confirmed by Southern blot analysis [30].

The volume of the dermis of E18.5 in wild-type and *Wnt11* knock-out mice embryos was analysed in paraffine sections after HE staining. We have used rectangular shaped dermis cross-section samples with 9 μm length and 20 μm width (thickness of the cross-sections). The height of the dermis was taken at 3 different locations (measuring the thickest, thinnest and medium points) on each cross-section and the average of these measurements was used to calculate the volume of the dermis in each slide (volume of dermis = 9×20×average height of dermis). For wild-type and *Wnt11* knock-out mice we have used for each 10 slides representing 3 mice embryos.

**Figure 1:**
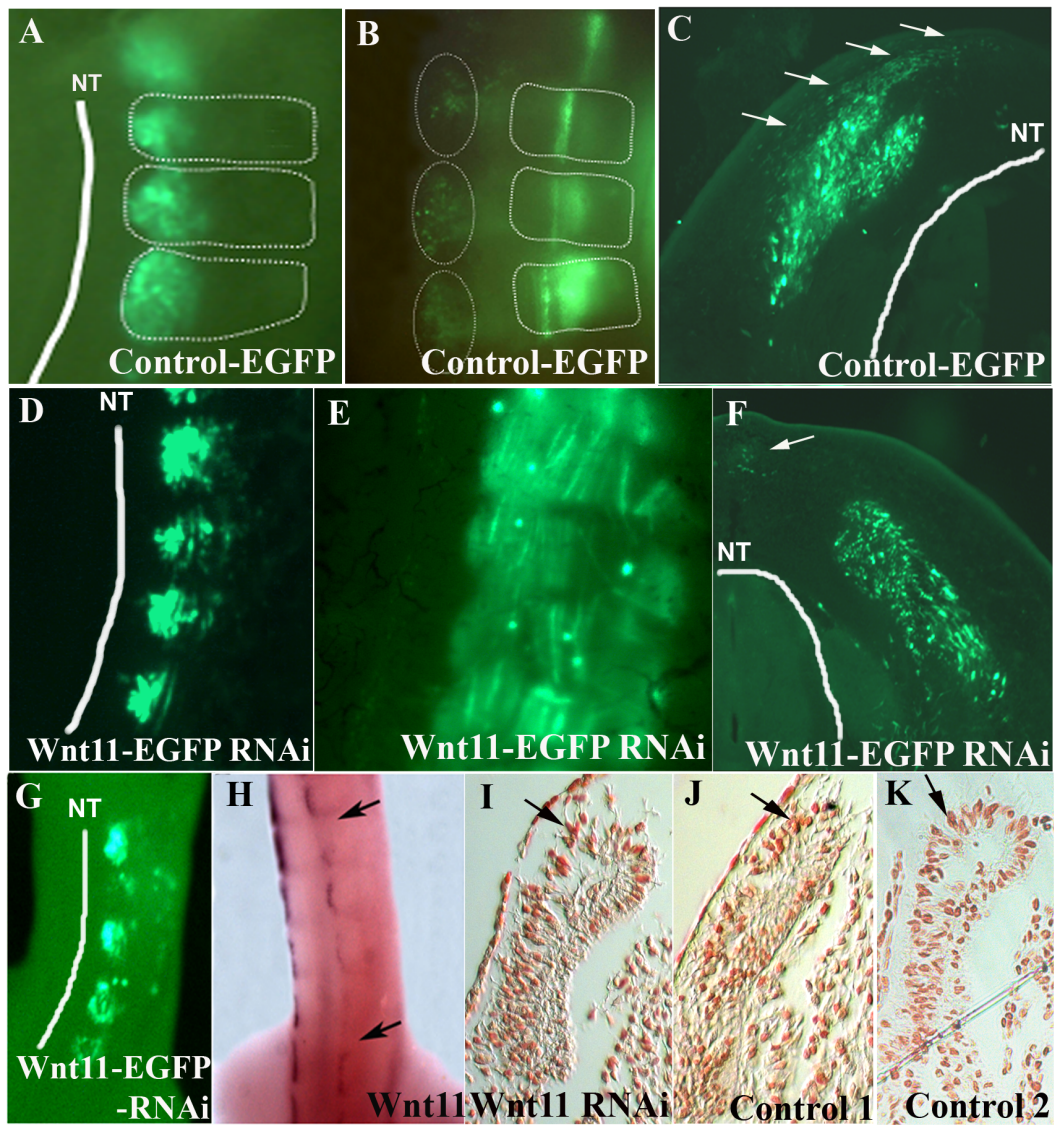
Murine dermis volume and hair follicles number comparison between *Wnt11* knock-out and wild-type mice. The diagram represents a graphical comparison of two averages with standard deviation bars of mice skin volume in sections of E18.5 mice wild-type and *Wnt11* knock-out mice. The results showed that the dermis volume in the sections of *Wnt11* knock-out mice was approximately 45% lesser when compared to the wild-type mice sections. The graphical comparison was performed in 20 sections after HE staining, 10 slides representing 3 mice for each genotype. The photos of the analysed sections were acquired at 40x magnification. For the graph representing the hair follicles number, the hair placodes on the back skin of the hybridized embryos for *mDermo-1* were counted in identical squares drawn on photos obtained using standardized magnification parameters. For this experiment, 6 *Wnt11* knock-out and 6 wild-type embryos were used. The diagram representing the graphical comparison of the two averages with standard deviation bars of hair follicles placodes number in mice embryos E14.5 hybridized for *mDermo-1*, shows a decrease of 35% in the number of hair follicles placodes in the mutant mice.

The average of dermis volume for wild-type and *Wnt11* knock-out mice embryos were plotted as a graph (Figure 1). The standard error was used for the error bar. The photos were taken at 40× magnification.

### qPCR

Mice dorsal skin samples derived from embryos of E12.5 and E16.5 were dissected and collected in screw cap tubes. Each tube contained a tissue quantity from which we could extract around 5 microgram of mRNA (approx. 500 mg of tissue). The dissected tissue was frozen immediately in liquid nitrogen.

mRNA from frozen tissue was extracted with Trizol (Invitrogen) and reversely transcribed with Quanti reverse transcription kit (Qiagen). For the qPCR, specific primer pairs (Table S2) for each analyzed gene were ordered from Invitrogen. 18s rRNA was used as reference gene. SYBR green qPCR master mix (Applied Biosystems) was used for the PCR reaction. Subsequent qPCR was carried out with DNA engine Opticon 2 Real-Time PCR Detection system (Biorad). The experiments were conducted with three independent sets of samples. Each PCR reaction was performed in triplicate. The threshold cycle (Ct) was acquired at the point where the fluorescence signal reached a value of 0.01. The result was presented as relative expression ratio (R) formulated as follows,
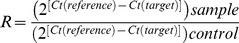



The data was introduced in REST software [42] for the calculation with the Pair Wise Fixed Reallocation Randomisation Test [43].

### Live-cell Imaging

Slices of chicken embryos (HH20) at the DML region were prepared under visual control with a binocular microscope. Specimens were aseptically cut into 175 μm thick transverse slices on a McIlwain tissue chopper, and then attached to collagen-faced (Sigma-Aldrich, C7661) glass coverslips (32 mm, Kindler, Freiburg, Germany) fixed by a plasma clot (Sigma, P3266) coagulated with thrombin (Calbiochem, 605157).

Time-lapse imaging was performed by the aid of confocal laser scanning microscopy (CLSM, Zeiss LSM 510) and 10x-Apochromate lens (Plan-Neofluar, NA 0.3) equipped with a laser module containing an Ar laser (488 nm) and a HeNe laser (543 nm). To maintain the incubation settings at 37°C and 5% CO_2_ on the microscope stage, a CTI controller 3700 digital, O_2_ controller, Temp control 37-2 digital, and the Incubator S_oxygen_ together with the heating insert P (Zeiss) are used. Typically, live-cell imaging of DML cell dynamics starts 24 hours after electroporation. Therefore coverslips are placed in a Rose chamber and covered with 2 ml phenol red-free nutrient medium to get best results in terms of brightness, contrast, acuity and resolution. To reduce phototoxicity of the cells and photobleaching of the FP-signal the excitation intensity was reduced to a minimum (488 nm, 2%, 543 nm, 11%) and images were taken every 10 minutes for a period of up to 10 hours [44].

## Results

### *Wnt11* Expressing DML is a Source of Dorsal Dermis Precursors

In the chicken embryo, local electroporation can be employed to perform gain and loss of function studies by either overexpressing or silencing genes of interest, respectively. This method is very convenient for spatially restricted manipulations of somatic cells, making the study of different developmental processes possible. In our work, we transfected the DML of embryos at stage HH14-17 with three different *Wnt11* RNAi producing plasmids and with scrambled DNA for control experiments and analysed changes in the development of the DML, the dermomyotome and its derivatives.

**Figure 2:**
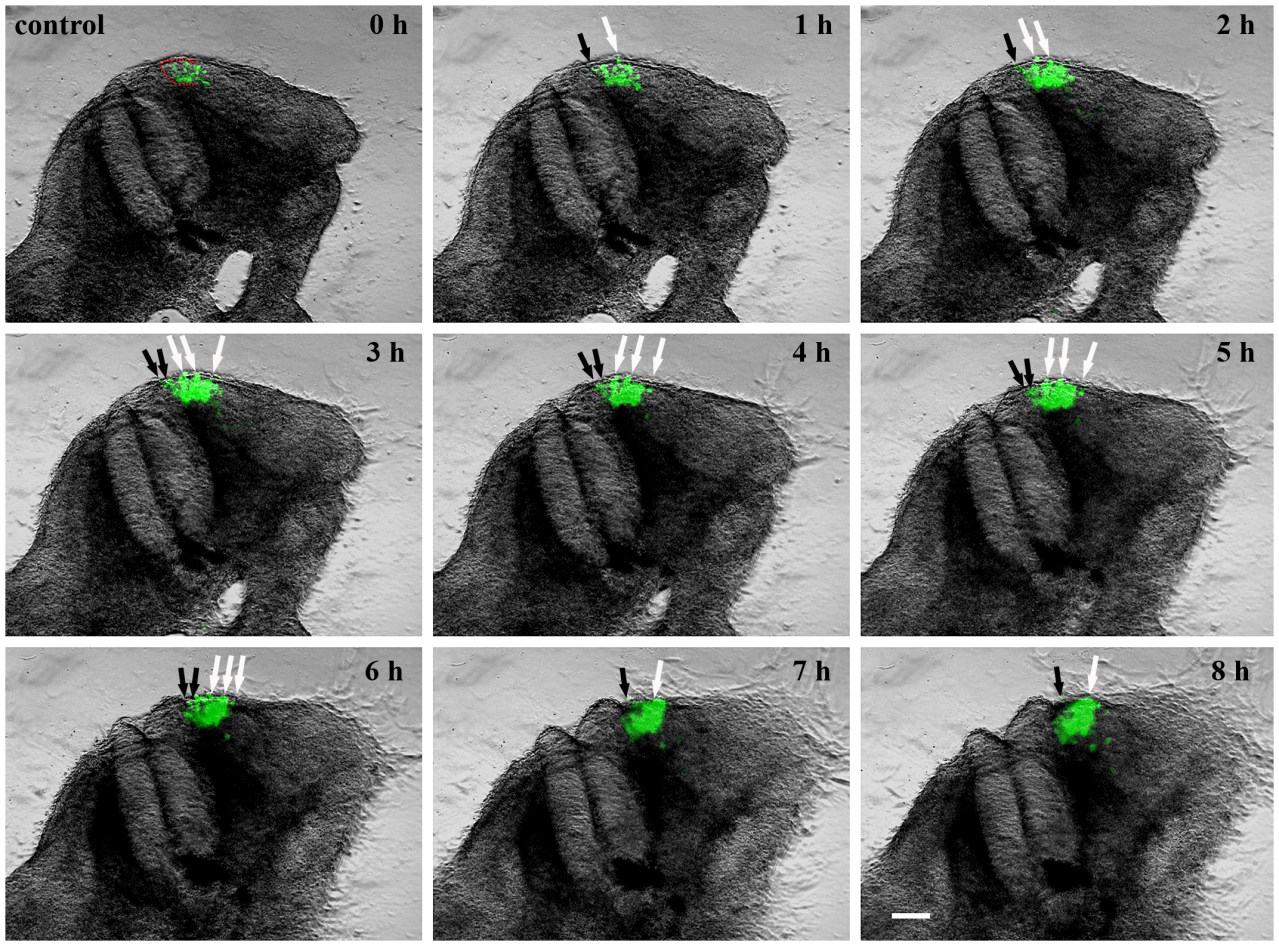
*Wnt11* expression in the DML is important for recruitment of dorsal dermal progenitors. A. EGFP-transfected DML of chicken embryo at stage HH18-19, 20 hours after electroporation. B. The embryo in A after 3 days of reincubation following electroporation. Note the presence of EGFP positive cells in the myotome and region of the future dorsal dermis (dotted circles). The white dotted lines squares delineate the somites. C. Cross-section of the embryo in B. EGFP-positive cells can be seen to be migrating into the subectodermal space overlying the spinal cord (white arrows). D. *Wnt11* RNAi on chicken embryo. DML of EFGP-*Wnt11* RNAi construct transfected chicken embryos at HH18-19, 20 hours after electroporation. E. After 3 days of reincubation following electroporation, the EGFP-*Wnt11* RNAi expressing cells are only to be found in the myotome in a disorganized manner, whereas EGFP-*Wnt11* RNAi positive cells are missing in the dorsal dermis anlage. F. In cross-section of the embryo in E, only very few cells (nearly undetectable) migrating from DML are present into the subectodermal space (white arrow) overlying the spinal cord (white line). White lines were traced along the neural tube for a better orientation. G. Targeting of the DML at stage HH14-17 by EGFP-*Wnt11* RNAi constructs after 24 hours reincubation and the corresponding *Wnt11* silencing as seen by ISH (area between black arrows in H). I. There is no evidence of increased cell death at the sites of EGFP-*Wnt11* construct transfection as seen by TUNEL staining. J. TUNEL staining of an electroporated embryo with scrambled DNA was used as control for our TUNEL assay. K. Represents the untreated control (Control 2) for TUNEL assay. Photos in I, J, K show the DML of the coressponding sections after TUNEL assay. Black arrows in I, J, K point towards Tunel-positive cells. NT: neural tube. DML: dorso-medial lip. Scale bar: 100 μm.

**Figure 3:**
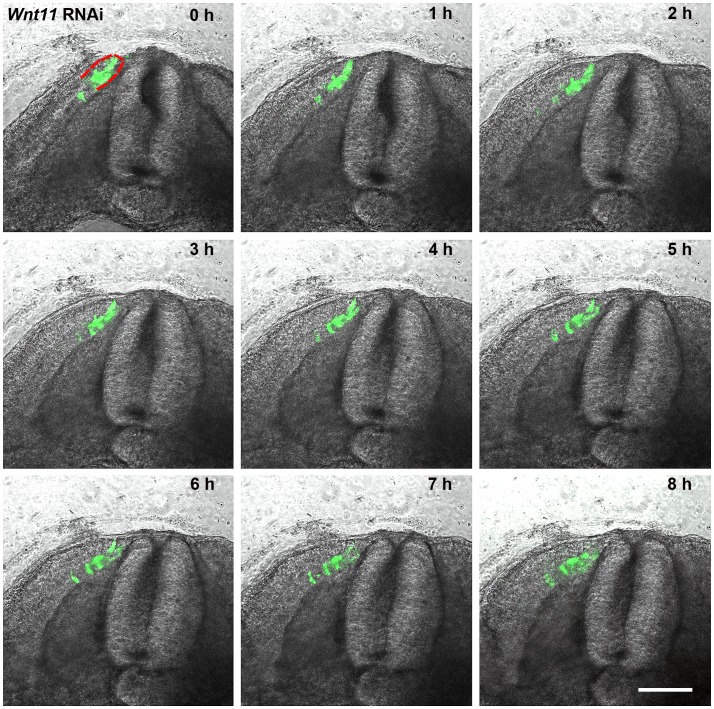
A series of 9 photos of a chicken embryo section electroporated with a control EGFP plasmid at the DML level and reincubated for 24 h. Each photo shows movement of cells in 1 h time interval. At 0 h the EGFP transfected cells are seen in the DML. At later time points, the dynamics of the transfected cells are visible, with cells moving to the subectodermal space above the neural tube (black arrows), and additionally migrating to the immediate neighbourhood of the neural tube (white arrows) and the myotome. These dynamic movements are characteristic of a normal distribution of DML cells, where DML cells migrate to the subectodermal space in order to form the dorsal dermis and to the myotome to form the muscle. The red outline indicates the DML. Scale bar: 100 μm.

**Figure 4:**
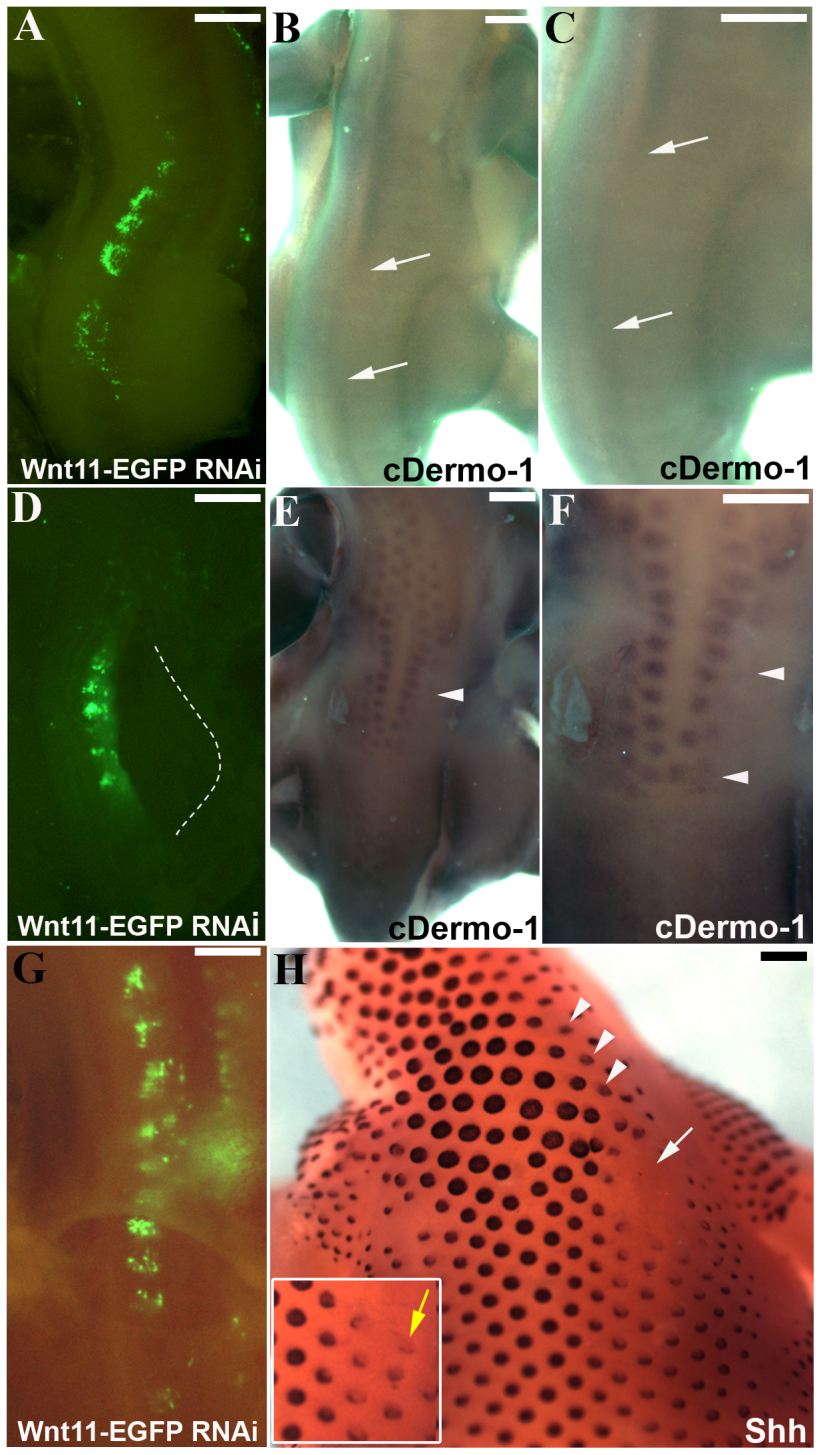
A series of 9 photos of a chicken embryo section electroporated with a *Wnt11* RNAi construct containing EGFP at the DML level, and than reincubated for 24 h. Each photo shows movement of cells in 1 h time interval. In contrast to the control experiment (Figure 3), the electroporated cells (green fluorescent) remain restricted to the DML or migrate into the myotome. No EGFP positive cells migrate towards the subectodermal space located above the neural tube or into the immediate neigbourhood. The absence of *Wnt11* in the DML thus results in a compromised EMT of the cells, which can only enter the myotome and no longer populate the subectodermal space above the neural tube. The red outline indicates the DML. Scale bar: 100 μm.

To confirm the efficacy of our in ovo electroporation protocol, we electroporated the DML of chicken embryos using an EGFP-containing control plasmid, reincubated and followed up the development of electroporated embryos. 24 hours later, EGFP was detectable in the DML (Figure 2 A) and after 3.5 days of reincubation following electroporation (HH29), EGFP positive cells were detected in the myotome, as already described [32]. Additionally, EGFP expressing cells were located above the neural tube where the future dorsal dermis will form (5 out of 5 embryos) (Figure 2 B–C). This was very clearly visible in our time-lapse experiments, when the EGFP-positive cells could be followed in their migration towards subectodermal space above the neural tube (seen in 5 out of 5 movies) (Figure 3/Movies S1 and S2). In another set of experiments, we silenced *Wnt11* gene expression in the DML by electroporation of shRNA-EGFP constructs directed against *Wnt11* mRNA (Figure 2 D). The expression of shRNA from these constructs was driven by either an H1 or a U6 promoter. Four *Wnt11* gene targeting constructs were individually tested for their efficiency and then used in a cocktail (see Material and Methods). This was followed by tracing the fate of EGFP-transfected cells while the silencing effect of the *Wnt11* RNAi vector was assessed by ISH analysis of the transfected embryos with mRNA probe specific for avian *Wnt11*. An effective silencing of the *Wnt11* gene expression was seen in the transfected region indicated by the EGFP signal (15 out of 20 embryos) (Figure 2 G–H). In the transfected embryos with *Wnt11* RNAi, after 3.5 days of reincubation time following electroporation (HH29), no EGFP-transfected cells were detected in the subectodermal region, however, many EGFP transfected myofibers were detected in the myotome. This was validated by our time-lapse experiments, when no EGFP-positive cells were observed to move towards subectodermal space overlying the spinal cord as usual. The migration into the myotome was unaffected (seen in 3 out of 3 movies) (Figure 4/Movies S3 and S4). The time-lapse experiment has been performed using two different constructs to interfere with *Wnt11*, *Wnt11* RNAi (Movie S3) and a DN-*Wnt11* RCAS construct (Movie S4). As already shown by Gros and colleagues in 2009, we also noticed the disorganized arrangement of the myofibers after interfering with Wnt11 signaling (11 out of 11) (Figure 2 E). This confirms the efficiency of our RNAi constructs when compared to a previously described phenotype [27].

No increase in cell death was detected at the site of *Wnt11* RNAi transfection in comparison with the control slides, evidenced by the TUNEL assay (Figure 2 I–K).

We can thus conclude that the DML does not only give rise to the myotome, but also to the subectodermal mesenchyme overlying the neural tube and that silencing *Wnt11* leads to an altered EMT of a subpopulation of DML-derived cells, resulting in an absence of subectodermal cells originating from the DML.

### Wnt11 is Required for Dense Dorsal Dermis

Once the silencing capability of our *Wnt11* shRNA constructs was established, we moved on to study the effects of *Wnt11* silencing on known dermal markers. The expression pattern of *cDermo-1* is noticeable from stage HH24 at low levels throughout the subectodermal mesenchyme of the trunk, being much stronger along the dorsal midline in the mesenchyme between dorsal ectoderm and neural tube. The expression intensifies at stage HH26 and is maintained until stage HH29. In older stages, the expression is strong in the mesenchyme of the nascent feather buds, underneath the ectodermal placodes. At stage HH36 the expression gets restricted to the mesodermal core of the feather buds [23].

**Figure 5:**
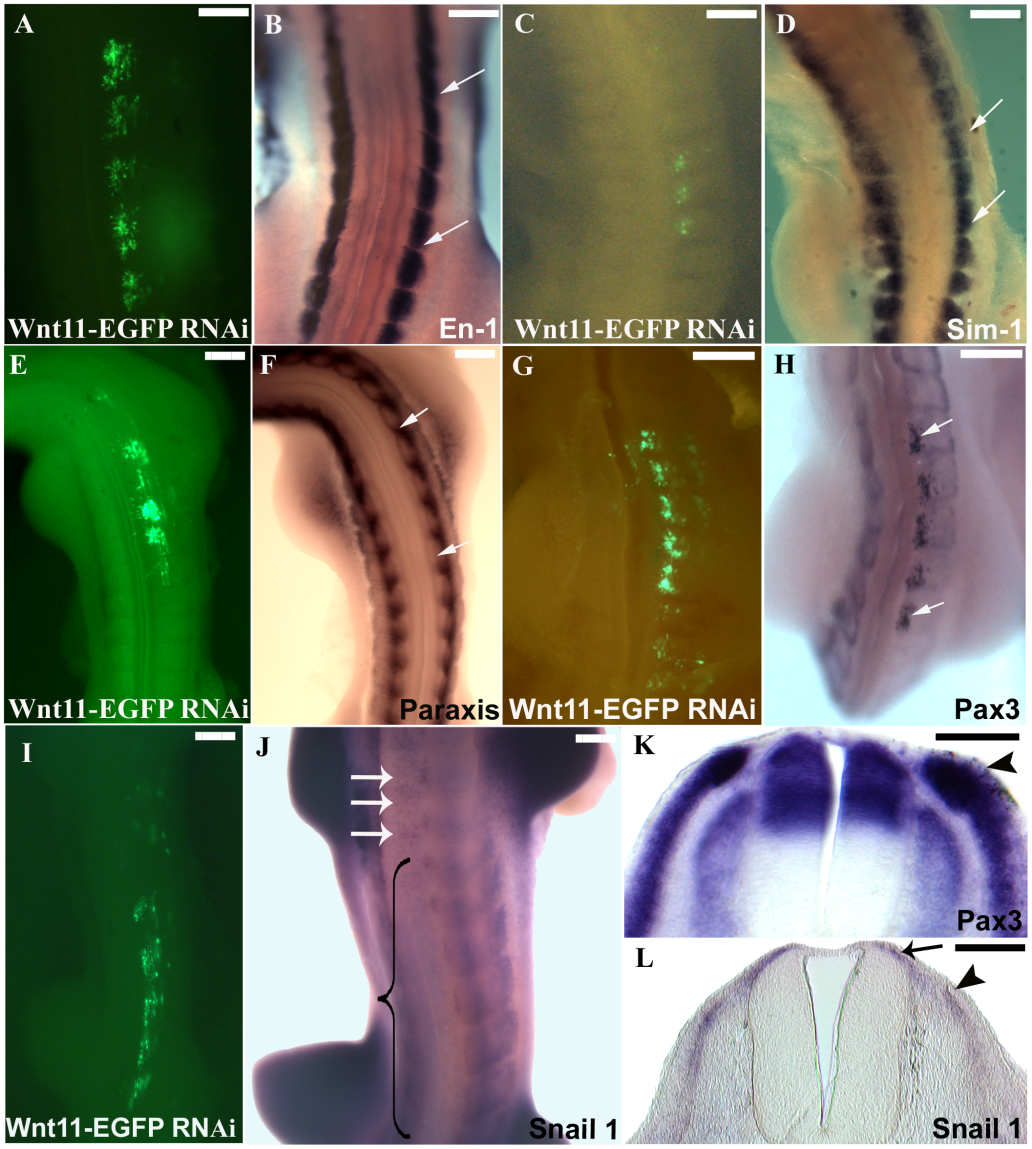
Decrease of dermal markers after *Wnt11* RNAi. A. Embryo showing the region of transfection after electroporation with EGFP-*Wnt11* RNAi. The embryo has been electroporated at stage HH14-17 and reincubated for 20 hours. B. The embryo in A after 2.5 days reincubation time following manipulation (HH28). Starting with stage 24, *cDermo-1* expression pattern can be found in the subectodermal mesenchyme of the trunk, and at stage 26 it is very strong along the dorsal midline. Following additional 2.5 days of reincubation after EGFP signal documentation for embryo in A, *cDermo-1* is remarkably reduced along the dorsal midline in the transfected region (area between white arrows in B). C. Higher magnification of the embryo in B. D. Electroporated embryo with *Wnt11* silencing construct at stage HH14-17 and photographed 20 hours later, at stage HH20. E. After longer reincubation periods following electroporation (4.5 days of the embryo in D), the embryo shows a retarded feather bud development as seen after *cDermo-1* ISH, which is expressed in this stage (HH31) in the mesenchyme of the nascent feather buds (the white arrowheads in E and F mark the missing row of feather buds on the manipulated side in D). F. Higher magnification of the embryo in E. G. Embryo showing the region of transfection after electroporation with EGFP-*Wnt11* RNAi at stage HH14-17 and photographed 24 hours later, at stage HH20. H. After 8 days reincubation for embryo in G after EGFP documentation (HH38) and hybridisation with a *Shh* probe we have noticed a retarded feather bud development as seen with *cDermo-1*. *In situ* hybridisation for *Shh* also reveals a changed morphology of the eventually formed feather buds, which are smaller and more flattened, with lower expression of *Shh* on the manipulated side (white arrow and arrowheads in H). The white square presents in a higher magnification the altered feather buds formation (yellow arrow). Scale bar: 100 μm.

In order to study the effects of *Wnt11* silencing on dermis and skin appendage development, the electroporated embryos were allowed to develop at least until the stage when the expression of the dermal marker *cDermo-1* starts or later to embryonic day 6 (HH29) when feather buds start to form. After 3.5 days of reincubation time following RNAi treatment, *in situ* hybridisation shows a significant downregulation of *cDermo-1* in the dorsal midline of the back (4 out of 5 embryos) (Figure 5 A–C). After a longer reincubation period following electroporation (when the embryos reached the stage HH31), we could observe the delay in formation of the first rows of feather buds in the manipulated area (3 out of 3 embryos) (Figure 5 D–F).

The retarded growth of feather buds was also demonstrated by analyzing older embryos (12 days), which were hybridized with an additional feather bud marker gene, *Shh*. This marker revealed the presence of much smaller and more flattened feather buds, expressing less *Shh* on the manipulated side with *Wnt11* RNAi (3 out of 3 embryos) (Figure 5 G–H).

These results support our hypothesis that Wnt11 plays a role in dermis formation. *Wnt11* silencing leads to a reduction of dense dermis, resulting in a reduced *cDermo-1* expression and also in a retarded and compromised formation of feather buds.

### Central and Lateral Dermomyotomal Identities are Maintained Following *Wnt11* Silencing in the DML

The dorso-medial lip and the central part of the dermomyotome are able to give rise to dense dermis competent for dermal condensations which will later bear feathers, while the lateral part is responsible for sparse dermis formation, characteristic of apteric regions. It is also known that while the dorsomedial lip of the dermomyotome expresses *Wnt11*, the central and the lateral compartments can be identified by their expression of two transcription factors, *En-1* and *Sim1*, respectively.

**Figure 6:**
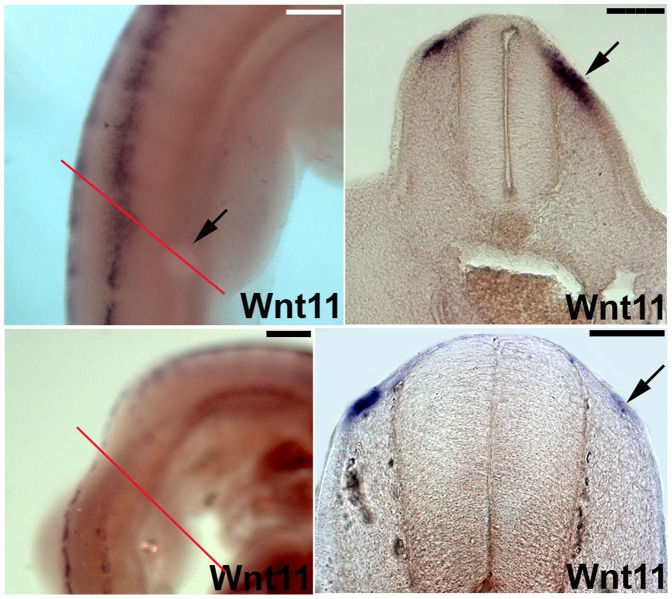
Effects of *Wnt11* silencing on dermomyotome. A, C, E, G and I. Chicken embryos electroporated with *Wnt11* RNAi at stage HH14-17 and after a reincubation period of 24 hours. B. The embryo in A (HH20) hybridized for *En-1,* shows that the central dermomyotomal compartment marked with *En-1* was unaffected in the electroporated area (the space between white arrows in B). D. The photo represents the embryo in C after ISH for *Sim-1* probe. The lateral dermomyotomal compartment marked with *Sim-1* (white arrows in D indicate the electroporated area) did not show any change in its expression after *Wnt11* silencing. F. ISH for *Paraxis* of the embryo presented in photo E. *Paraxis* transcripts seems not to be altered following *Wnt11* silencing in the DML (area between white arrows in F). H. The dermomyotomal marker *Pax3*, in contrast, is significantly upregulated at the site of *Wnt11* RNAi transfection (area between white arrows in H). K. The cross-section through the embryo in H in the manipulated area shows a strong upregulation of *Pax3* in the DML, while the DM remains normal when compared to the control side. I. Electroporated embryo at stage HH14-17 with *Wnt11* RNAi and after 24 hours reincubation (HH19). J. Hybridized embryo from photo I for *Snail1* probe. At stage HH20 the EMT has already started, and the *Snail1* expression can be seen in the myotome, dermomyotome, sclerotome and in the space above the neural tube (dermal progenitor cells). Whole-mount ISH of the embryo electroporated with *Wnt11* RNAi shows a decreased *Snail1* expression (the region in the bracket), while the white arrows point towards the normal expression of *Snail1* above the neural tube (dermogenic progenitors) at untreated levels. L. Section through the embryo in J in the affected region shows a decreased *Snail1* expression in the DML (black arrowhead) and above the neural tube (black arrow). Scale bar: 100 μm.

To check if these compartments are affected following silencing of *Wnt11* in the DML, we performed ISH for *En-1* and *Sim1* on *Wnt11* RNAi-treated embryos. None of the electroporated embryos analyzed for these markers showed any changes in the expression of the above markers (10 embryos analysed for each marker) (Figure 6 A–D).

The results revealed that the silencing of the *Wnt11* gene in the DML did not affect other parts of the dermomyotome. This is probably due to the fact that silencing of *Wnt11* in the DML affects only the dorsal dermis, whereas the central and the lateral regions of the dermomyotome and the tissues that arise from it remain unaffected by this manipulation and develop independently of the DML.

### Effect of *Wnt11* Silencing on Genes Expressed in Dermomyotome

*Paraxis* [45] and *Pax3* [46], [47] are further transcription factors expressed in the dermomyotome [48]. *Pax3* is a progenitor cell marker for the entire premyogenic and myogenic lineage and also plays a role in maintaining the dermomyotomal cells in an uncommitted state. To assess if *Wnt11* silencing has an effect on the epithelial state and commitment of the dermomyotome, we looked for possible alterations in expression of *Pax3* and *Paraxis* after electroporation. ISH for *Paraxis* did not reveal any alterations in its transcript distribution (40 embryos out of 40) (Figure 6 E, F). In contrast, we observed a strong upregulation of *Pax3* (Figure 6 G, H), strictly corresponding to the EGFP signal in the electroporated embryo. Section through one of the embryo hybridized with *Pax3* probe shows a strong upregulation of this gene in the DML, but no changes in the structure of the dermomyotome (Figure 6 K). Electroporation of scrambled shRNA constructs affected neither *Pax3* nor *Paraxis* expression. These results show that the cells which would normally adopt a dermogenic fate remain uncommitted, as shown by the strong upregulation of *Pax3* (25 embryos out of 25). This is an indication that DML cells require Wnt11 for unfolding their future fate as dermogenic cells.

*Snail1* has been described in context of deepithelialisation. Loss-of-function studies for *Snail1* in chicken embryos resulted in delayed EMT with the maintenance of the epithelial structure of the dermomyotome [29]. Following initiation of EMT in the dermomyotome, the *Snail1* positive cells can be detected in the myotome, dermomyotome, sclerotome as well as in the subectodermal space above the neural tube (dermogenic progenitor cells). We analysed the effect of *Wnt11* RNAi on the expression of *Snail1* in the DML following electroporation. A remarkable decrease in the *Snail1* expression was observed after *Wnt11* silencing in the DML of the dermomyotome (8 out of 9 embryos) (Figure 6 I, J, L). Our results show that Wnt11 is required for the induction of dermal fate in the DML progenitors.

### Wnt11 is Regulated by BMP2 Signaling in the Dermomyotome

**Figure 7:**
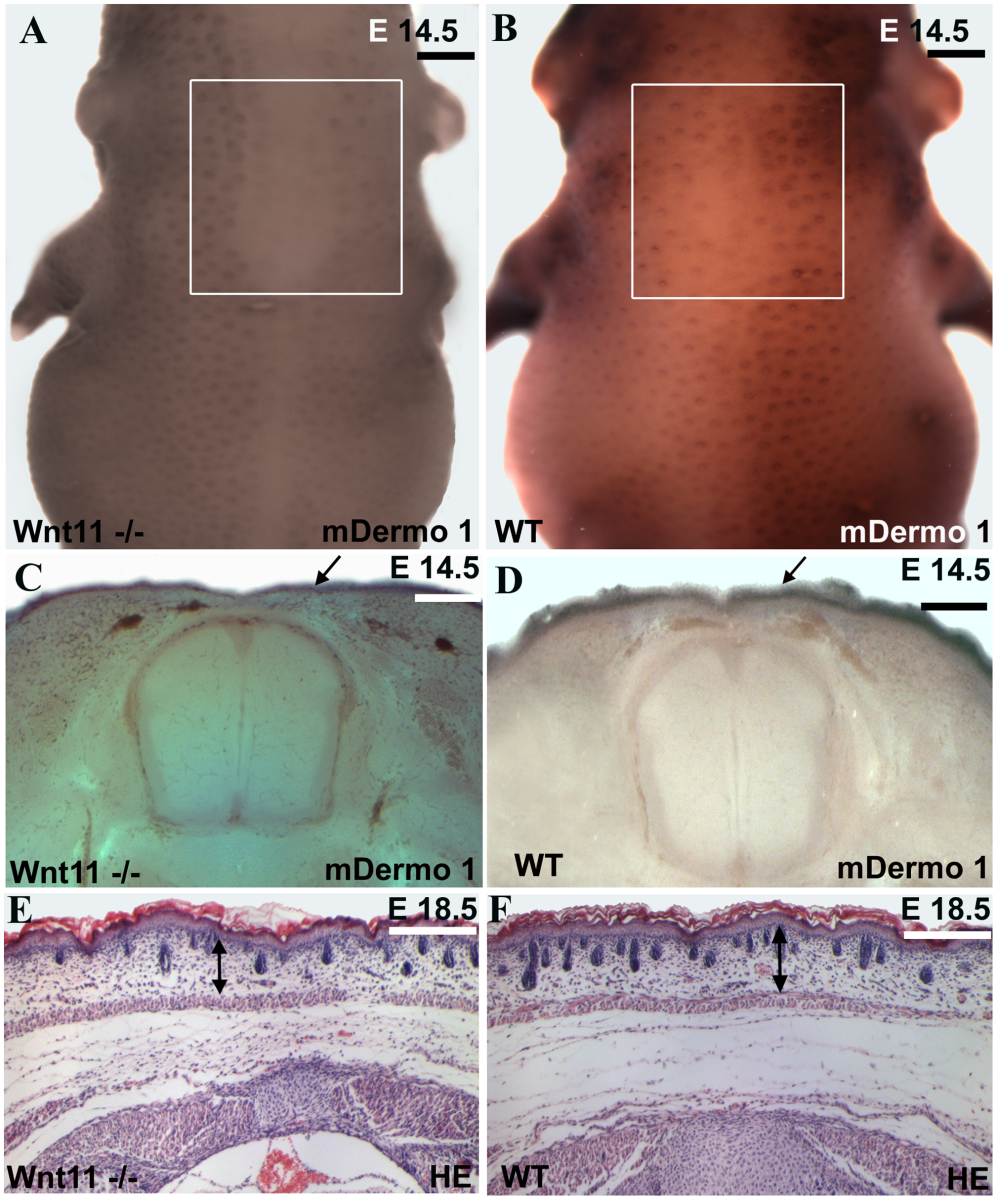
BMP2 signaling controls *Wnt11* expression in DML. A, B. BMP2 beads implanted into somites of chicken embryos (black arrow in A) lead to an upregulation of *Wnt11* as seen by ISH in vibratome sections (black arrow in B; section level indicated by red line in A). C, D. In contrast, grafting of Noggin cells (BMP antagonist) lead to a downregulation of *Wnt11* transcripts on the operated side (C) also visible in vibratome sections of the same embryo (black arrow in D; section level indicated by red line in C). Scale bar: 100 μm.

Previous findings in our group have shown that BMPs promote dermogenesis [25]. Implantation of beads soaked in different concentrations of purified BMP2 protein in chicken embryos of stage HH13-17 upregulates *cDermo-1* expression. On the other hand, BMP-antagonist Noggin is able to downregulate *cDermo-1* when applied at appropriate stages of dermal development. To test our hypothesis concerning the role of Wnt11 during dermis development, we were interested to see if *Wnt11* expression would also respond to dermogenic and anti-dermogenic signals in a similar pattern (Figure 7). Using BMP2-coated beads, we could show that the *Wnt11* expression domain could be broadened (5 out of 6 embryos), while Noggin, a BMP inhibitor, was able to downregulate *Wnt11* expression in the somites (6 out of 6 embryos) (Figure 7 A–D). These experiments reveal that Wnt11 is regulated similarly to cDermo-1, as both respond to BMPs and Noggin in a similar fashion thus supporting our hypothesis that Wnt11 plays a role in dermis development.

### Compromised Hair Follicles and Dermis in Knock-out Mice for *Wnt11*

**Figure 8:**
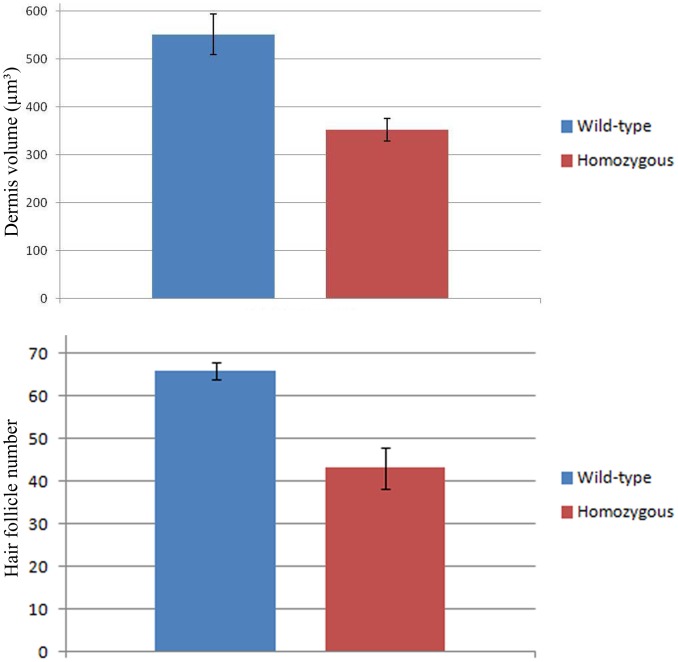
Dorsal dermis is decreased in dermis thickness and number of hair follicles in the *Wnt11* knock-out mice. A, C, E. Homozygous *Wnt11* knock-out embryos. B, D, F. Wild-type embryos. Embryo in A represents a homozygous *Wnt11* knock-out embryo of embryonic day E 14.5. In the dorsal mid-line region of this embryo a large area devoid of hair placodes is detectable. The white square shows a decrease number of hair placodes in comparison to the WT embryo of similar developmental stage (B). Section through the embryo in A shows that the *cDermo-1* signal is diminished with corresponding decrease in dermal thickness in the mutant (black arrow in C) as compared with a similar section of the same magnification of a WT embryo (black arrow in D). Hematoxylin-Eosin staining in paraffin sections of knock-out mice of E 18.5 shows a remarkable decrease in dermis thickness (45%), as well as a reduced number of hair follicles (35%). (E) In comparison with a Hematoxylin-Eosin treated section of a WT mouse of the same developmental stage (F). Double-headed arrows in E and F show the remarkable difference in dermis thickness between the mutant mouse embryo as compared to the normal thickness of dermis in a wild-type embryo (photographs were taken at the same magnification). The sections were taken at the trunk regions of the mice. The analysis was restricted to the same anatomical area of the dorsal skin for all embryos investigated. Scale bar: 100 μm.

To validate our loss-of-function and gain-of-function findings for *Wnt11* from chicken embryos, we went on to look for alterations also in the dermis in *Wnt11* knock-out mice [30]. In situ hybridizations performed for *Twist2* (*mDermo-1*) on *Wnt11* mutants of E14.5 revealed a large area in the dorsal midline region of homozygous mutants devoid of hair placodes in comparison to the wild-type embryos of the same developmental stage (Figure 8 A–B). The hair placodes on the back of the hybridized embryos were counted in identical squares drawn on photos obtained using standardized magnification parameters. We could document a decrease of approximately 35% in hair follicles number in homozygous mutant embryos as compared to the wild-type littermates (6 embryos analyzed each for homozygous and wild-type) (Figure 8 A–B). A graph of hair follicles number comparison between *Wnt11* mutants and wild-type mice is presented in Figure 1. By inspection of vibratome sections of these embryos that had previously undergone whole mount ISH, a decrease in the depth of the *Twist2* (*mDermo-1*) expression domain of the mutants in comparison to the wild-type embryos became evident (Figure 8 C–D).

Additionally, the skin from wild-type mice and homozygous mutants of *Wnt11* of day E 18.5 was compared in paraffin sections. Hematoxylin-Eosin staining of these sections revealed a noticeable decrease in size of the dermis layer in *Wnt11* homozygous mice compared with their wild-type littermates. Upon quantification, we observed a significant decrease in dermal volume of about 45% (Figure 8 F, Figure 1) associated with a reduced number of hair follicles (35% decrease) in the trunk region of homozygous *Wnt11* mutants in comparison to the wild-type embryos (10 slides representing 3 mice embryos were analysed for each genotype) (Figure 8 E, Figure 1).

### Expression Analysis of Knock-out Mice for *Wnt11* by qPCR

Further quantitative analyses of the expression of regulators of dermis development were performed using qPCR (Real-Time PCR).

**Figure 9:**
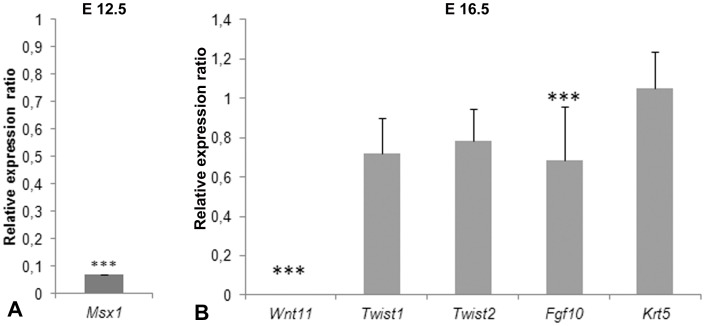
Relative expression of dorsal skin development-related genes in *Wnt11* knock-out mice and wild-type. A. At stage E12.5, *Msx1* shows much less expression in mutant mice than wild-type mice. (***:P<0,001). B. At stage E16.5, *Wnt11* expression in *Wnt11* knock-out mice is undetectable compared to wild-type mice. *Fgf10* has less expression in the homozygous mice. *Twist1*, *Twist2* and *Krt5* expression does not show significant differences between wild-type and homozygous animals.

An early marker of dorsal dermogenesis has been considered to be the homeobox gene *Msx1* which at the same time also stains cartilage precursor cells of the vertebral spinous process in chicken embryos, although at earlier stages [49]. *Msx1* has been shown to control the process of differentiation in the dorsal-most dermis lying above the neural tube. During their migration from the DML towards the subectodermal space, precursor cells of the dorsal dermis express *Msx1* [12]. At stage E12.5 when *Msx1* is expressed in the subectodermal space (the place where in the chicken *cDermo-1* is expressed in a similar developmental stage) above the neural tube, we found that *Msx1* expression was considerably less (P<0,001) in the *Wnt11* mutant mice than in the wild-type animals (Figure 9). At this stage, *mDermo-1* expression has not yet started medially in the space above the neural tube. Therefore the downregulation of *Msx1* at E12.5 in the mouse is comparable with the downregulation of *cDermo-1* at day 6.5 of embryonic development in the chicken embryo. Unchanged expression levels of *mDermo-1* were not surprising, considering that the source of the analysed mice skin pieces was the most dorsal (medial) back where expression of *mDermo-1* had not yet started.

In stage E16.5, skin samples processed for qPCR showed a complete absence of *Wnt11* expression in the *Wnt11* knock-out mice. Compared to the wild-type animals, *Fgf10* also showed a lower expression in homozygous mice. *Fgf10* is expressed in the dermis, with a role in mesenchymal-epithelial signaling during skin development [50]. In the chicken, *Fgf10* is expressed in the dermis of nascent feather primordia [51]. and *cDermo-1* acts upstream of this early dermal marker [24]. The transcription factor *Twist2* (*mDermo-1*) is expressed in dermis during mouse embryonic development [22] and acts redundantly with *Twist1* [52]. There was no significant change of expression levels for *Twist1* and *Twist2* (*mDermo-1*) comparing the knock-out and wild-type mice. At E16.5, we also used the dorsal dermis from the midline of the trunk region. At this time, the expression domain of *Twist2 (mDermo-1),* has already spread dorsally. However, when performing the qPCR, we normalized the expression analysis for the amount of tissue. As shown in Figure 8C and D, there are fewer cells in the dorsal domain. So, the expression levels of *Twist2* were not reduced, but the number of cells expressing *Twist2 (mDermo-1*) were. However, for induction of the skin appendages the density of dermis is critical.

No change was observed also in the case of *Krt5* between knock-out and wild-type mice. Krt5 is a type II cytokeratin produced in mitotically active keratinocytes, in the basal layer of the epidermis, that maintain the proliferation potential of this layer [53].

## Discussion

Dorsal skin morphogenesis can be divided into three successive phases during embryogenesis. In the first phase, the dermogenic progenitors delaminate from the dermomyotome in an epithelio-to-mesenchymal transition (EMT) and migrate under the ectoderm to form dense dermis. This initial event is followed by a cross-talk between the dermal condensations and the overlying epidermis leading to the formation of skin appendage primordia (epidermal placodes) and the final and third phase consists of the organogenesis of the appendages. Although several publications have arisen over recent years aiming to dissect the mechanisms regulating the second and third phase of skin morphogenesis [16], [54], [55], little is known about the signaling and the molecular players involved in the initial phase of dermis development. In our study, we address the first phase of dermogenesis and only use the formation of skin appendages as a readout for the successful formation of dense dermis.

In avians, the dorsal dermis is derived from the medial part of the somitic dermomyotomes which is responsible for the formation of the dense dermis competent for feather bud induction, while the lateral compartment is the source of sparse dermis specific for apteric regions [16]. Based on its embryonic expression (Figure S1) in the dorsomedial lip of the dermomyotome and subectodermal mesenchyme, *Wnt11* was proposed as a candidate gene involved in the formation of dermis [26]. At the same time, Wnt11 has been extensively linked to morphogenetic movements such as cell migration [56] and convergent-extension movements [57] during development. Although *Wnt11* is not restricted to the dorsal dermis progenitor cells, but throughout the nascent dermis, the avian dermomyotome represents a region of special interest which lends itself readily to the electroporation. In our study, we found that knocking-down *Wnt11* affects the patterning behaviour of somite-derived dermal progenitor cells leading to a reduced expression of the early dermal marker *cDermo-1*) [23].

We could furthermore show that *Wnt11* is also regulated in a manner similar to *cDermo-1*. Our results obtained in chicken embryos are corroborated by our analysis of murine *Wnt11* mutants which show thinner dermis than their wild-type littermates, and impaired hair follicle development in the trunk region. In combination with previous studies, our results pinpoint the role of Wnt11 in dermis development, in particular that of dense dermis.

The origin of the dorsal dermis directly above the neural tube in mouse and chicken has been a matter of controversy as its origin was initially proposed to lie in the sclerotome as well as in the dermomyotome [15]. This idea arose from the fact that progenitor cells for dermis and cartilage, both expressing *Msx1*, are co-localized above the dorsal neural tube [12]. Using chicken-quail graft experiments, the sclerotomal origin of the spinous processes and neural arches and the dermomyotomal origin of the dorsal dermis progenitor cells were shown, these processes being temporally separated. The dermomyotomal cells will deepithelialize and migrate towards the dorsal neural tube to form dermis only after chondrogenesis of the vertebral arches has occurred [49]. In our study, we found that at the time when the dorsal dermis is formed in the mouse (E12.5), *Msx1* was expressed to a lesser extent in the progenitor cells for the dorsal dermis in *Wnt11* mutants as compared to the wild-type embryos indicating that the cells now present in the dorsal area had an identity different from the ones that would normally have formed the dorsal dermis. This explains why there was no decrease in the expression of *mDermo-1 (Twist 2)*, because the medial dermis was likely replaced by dermal progenitors from further lateral that also express *mDermo-1.*

Experiments in the chicken involving DiI labeling and grafting further endorse the dermomyotomal origin of dorsal dermis [15] and we can confirm these findings by our experiments. Employing cell lineage tracing techniques with electroporation of EGFP reporter plasmid into the DML, we show here that the marked EGFP-expressing cells not only move ventrally to enter the myotome, but also delaminate and migrate dorsally above the neural tube under the ectoderm in the dorsal dermis anlage. Sclerotomal cells expressing EGFP were never found in our cell tracing experiments indicating that only the dermomyotomes were targeted. Thus using a different cell labelling technique and live imaging, we show that the DML is a source of dorsal dermal precursors. Cells from the DML delaminate and migrate to the subectodermal region above the dorsal neural tube to form the future dense dermis capable of skin appendage induction. This technique has revealed the presence of dorsal dermis precursors precisely from the DML using live-imaging for the first time to our knowledge.

The dorsomedial lip (DML) has been described to contain a complex population of multipotent stem/progenitor cells [58]. RNAi silencing of *Wnt11* in the DML prevented the cells from migrating from the DML towards the subectodermal space. In contrast to the dermogenic lineage, the silencing effect of *Wnt11* in the DML does not appear to quantitatively affect the migration of myogenic precursors into the myotome. We regularly find in our experiments EGFP expressing myoblasts and myofibers in the myotome following *Wnt11* shRNA treatment in comparable numbers to the normal situation, as has previously been reported by others [27]. This is in accordance with data showing that although myotome cells do form after *Wnt11* silencing, their arrangement is altered [27] which served in our study as a positive control for the efficacy of our silencing constructs.

The earliest known marker of dermal precursors, *cDermo-1* has been shown to be expressed from HH24 onwards in the subectodermal space of the dorsal midline region of chicken embryos [23]. It was later demonstrated that BMPs influence the expression of *cDermo-1* in a positive manner promoting dense dermis formation and epidermal appendage formation in a distinct and time-dependent manner [25]. The application of exogenous BMP proteins leads to local upregulation of *cDermo-1*, while the BMP antagonist Noggin results in a downregulation of *cDermo-1* transcripts and a lack of dermal tissue [24]. Based on the hypothesis that Wnt11 may play a role in dermis development, we tested the effect of BMPs and Noggin on *Wnt11* expression. The parallel effects observed on *Wnt11* and *cDermo-1* expression following application of BMP and Noggin further strengthen the hypothesis that Wnt11 plays a role in dermogenesis.

We observed a loss of *cDermo-1* following *Wnt11* RNAi in the region where dorsal spinal pterylae are known to develop. Silencing of *Wnt11* in the DML did not affect more laterally located regions of the dermomyotome also known to contribute to dermis formation. This was reflected by an unchanged expression pattern of *En-1* and *Sim1* following *Wnt11* silencing in the DML, implying that Wnt11 does not influence the formation of these dermomyotomal compartments and showing that the derivatives of the dermomyotome maintain a topographical relationship with their epithelial ascendants [4].

In our experiments, silencing of *Wnt11* in the DML did not alter the state of the already epithelial dermomyotome as reflected by the unaltered *Paraxis* expression and size of the dermomyotome. *Paraxis* is known to maintain the epithelialisation of the dermomyotome [45]. Another transcription factor, *Pax3*, known to keep the dermomyotomal cells in an undifferentiated state, is upregulated after RNAi of *Wnt11*.

The dorsomedial lip of the dermomyotome not only provides post-mitotic myoblasts for the myotome, but also contributes to the medio-lateral growth of the dermomyotome [59]. It has been reported earlier that *Wnt11* acts as an epithelialization factor for the medial dermomyotomal lip [17]. Our results, obtained by a loss-of-function approach for *Wnt11*, do not support this idea. Dermomyotomes were normal in appearance and size in all of our experiments. Moreover, we even observed a decrease in the deepithelialization marker *Snail1* following *Wnt11* RNAi. The release of dermal and myogenic progenitors from the dermomyotome is preceded by an epithelio-mesenchymal transition (EMT) event that ensures a timely and ordered allocation of these cells into their respective topographical destinations. Our data are more consistent with the observation that after *Wnt11* RNAi, the EMT marker *Snail1* was downregulated. This would indicate that the processes in which this gene is involved were disturbed, and as a consequence, the cells are not realizing their normal fate decision, as shown by the strong upregulation of *Pax3* in the DML. We could clearly visualize the process by using live-cell imaging, where the distribution of the DML cells in the dermal and myogenic compartments are observed as expected using control constructs. FGF8 signaling from the nascent myotome has been shown to act via the MAPK/ERK pathways leading to the activation of the downstream transcription factor *Snail1* in the dermomyotome [29]. The *Snail* family of genes controls EMTs in many cellular contexts both during embryonic development as well as in cancer metastasis [60], [61]. Ectopic expression studies of *Snail* in the chicken hindbrain show that the induction of EMT leading to neural crest formation is a conserved role associated with the Snail family of zinc-finger transcription factors [62]. In the early chicken embryo, *Snail1*, and not *Snail2* is expressed in the somites [63]. These findings suggest that Wnt11 functions in the opposite way to the previously reported one in the dermomyotome, as we observed that the absence of *Wnt11* rather serves for the maintenance of the epithelial dermomyotome by a decrease in the EMT as evidenced by the downregulation of *Snail1* in our study. Live-cell imaging performed on sections of electroporated chicken embryos, treated with a construct for *Wnt11* silencing or treated with a DN-*Wnt11*-construct, shows a restriction in the distribution of the cells from DML and their absence from the subectodermal space above the neural tube. The migration into the myotome where cells keep close contact to the dermomyotome was not affected. Thus, it appears that dermis progenitor cells cannot undergo EMT from the dermomyotome.

Wnt11 has previously been reported to be involved in the cell shape changes associated with convergent extension movements during embryonic development [57], [64]. Furthermore, acting via the evolutionary conserved PCP pathway, Wnt11 ensures the proper orientation of the early myofibers in the myotome [27]. *Wnt11* is an orthologue of *Wnt11-R* in *Xenopus*. It is known from *Wnt11-R* mutants that Wnt11 is required for somitic precursor migration towards the dorsal fin [56]. This dorsally directed migration in anamniotes may have set the grounds for the formation of dorsal dermis in amniotes during evolution, thus employing the same set of molecules in new contexts. It appears that the observed effects on *Wnt11* silencing can be attributed to an endogenous role of Wnt11 in an autocrine/paracrine mechanism in the silenced cell population in the dermomyotome as shown by our work here and other groups [27]. Taken together, these results confirm our hypothesis regarding the direct relationship between *Wnt11* and dense dermis formation. Our analysis of *Wnt11* knock-out mice supports our results in the chicken as we observed a significant decrease in the dorsal dermal volume and reduction in hairs in the mutants. Furthermore, our interpretation of the results is also supported by quantitative PCR showing that the dermally expressed genes *Fgf10* and *Msx1* are decreased in mutant embryos in comparison to the wild-type.

Although there appear to be differences between mouse and chicken regarding the control of *mDermo-1* (*Twist2*), this may be partially attributable to the different experimental approaches in the two species. Experimental work in the chicken targets individual cells at a particular time point, while in the mouse, the system has a long time to adapt to the loss of *Wnt11* and other genes or mechanisms may compensate for this. This is further supported by the fact that *Msx1* is strongly downregulated in the mutant at E12.5 indicating that the migration from the DML is initially impeded also in the mouse. The cells that successfully make their way to the dermis do not downregulate *mDermo-1*, but there are fewer cells, which can be seen by ISH. However, it will be difficult to detect in qPCR, as this approach is normalized for the loaded amount of tissue. So, the number of cells expressing *mDermo-1* were reduced, but the expression levels of each cells was not. However, for induction of the skin appendages the density of dermis is critical.

In summary, our results show that Wnt11 is required for the formation of dense dorsal dermis which is capable of forming cutaneous appendages. In the absence of *Wnt11*, dermal progenitor cells fail to acquire their fate and consequently fail to populate the subectodermal space overlying the neural tube. The downregulation of *Snail1* in the dermomyotome after *Wnt11* silencing argues for a compromised EMT at the DML. Successfully electroporated cells can however still be found in the myotome. Interfering with EMT leads to a decreased number of dermal precursors that can reach the subectodermal space resulting in a lower cell density of the dermis thereby diminishing their effectiveness to form feathers [15]. We suppose that these cells remain either in the DML in an undifferentiated state, as shown by strong upregulation of *Pax3* in the absence of *Wnt11* or enter by default into the myotome. Most EGFP-positive cells are found in these two locations. However, as none of these structures seem to have increased in size, an adjustment of proliferation could be simultaneously involved.

To conclude, our results show that Wnt11 is necessary for dermomyotomal cells in acquiring the dermogenic fate and migration towards the dermal anlage under the ectoderm. Thereby, we could experimentally substantiate the hypothesis based on its expression pattern that *Wnt11* is essential for dermis development and effective cutaneous appendage formation.

## Supporting Information

Figure S1 **Expression of** ***Wnt11.*** A. Whole-mount ISH for *Wnt11* at stage HH18. E. Cross-section through the embryo in A. Expression of *Wnt11* is limited to the dorso-medial lip of the dermomyotome (black arrowhead). C. Whole-mount ISH for *Wnt11* at stage HH20. As the embryo develops, the expression domain extends more caudally, retaining its prominent expression cranially. F. In the cross-section of the embryo in B, we can observe a stronger expression in the dorso-medial lip (black arrowhead), some cells *Wnt11* positive are located between the dorso-medial lip and the neural tube (black arrow) and on top of the neural tube (red arrowhead). C. The whole-mount ISH for an HH22+ stage embryo shows a prominent expression of the *Wnt11* transcripts, maintaining its cranio-caudal gradient. G. The cross-section through the embryo HH22+ reveals a broader area of the *Wnt11* gene expression, including the area between the DML and neural tube, the anlage of the future dorsal dermis (black arrow) and above the neural tube (red arrowhead). Its expression pattern in the dorso-medial lip is also increased. D. Whole-mount expression of a stage HH24 embryo. The strong expression of the *Wnt11* transcripts becomes obvious. H. The cross-section of the HH24 stage embryo hybridised for the *Wnt11* gene shows clearly the large area of cells positive for *Wnt11*, including the subectodermal space (black arrow and black arrowhead) and also an increased expression on top of the neural tube (red arrowhead), which later on will form the dorsal dermis. All the sections showed in this figure were performed at the interlimb level. Scale bar: 100 μm.
(TIF)

Table S1 **Selected nucleotide sequences for silencing of** ***Wnt11*** **mRNA.** This table presents the four selected target sites. The first target site starts at +217 nt, the second target site starts at +326 nt, the third target site starts at +761 nt and the fourth target site starts at +1034 nt. The forward and reverse strands of the shRNA inserts were designed as presented in this table.
(PDF)

Table S2 **Primers used for qPCR.** Specific primer pairs used for qPCR of the genes analysed. 18sRNA was used as a reference gene.
(PDF)

Movie S1 **Time-lapse of control EGFP electroporated embryo 1.** 175 μm thick slice of a HH20 embryo electroporated with a control *EGFP* plasmid (24 hours after transfection). The dynamic migration of the cells from the DML towards the subectodermal space and above the neural tube were observed. The migration was followed for 10 hours.
(MP4)

Movie S2 **Time-lapse of control EGFP electroporated embryo 2.** Similar section as described in Movie S1 reconfirms the high number of the cells moving from the DML towards the subectodermal space and above the neural tube.
(MPG)

Movie S3 **Time-lapse of** ***Wnt11*** **RNAi electroporated embryo.** 175 μm thick slice of a HH20 embryo electroporated with *Wnt11* RNAi plasmid (24 hours after transfection). Very few cells migrating towards the subectodermal space and above the neural tube were observed in comparison with the control time-lapse experiment. The migration of the EGFP –positive cells to the myotome is not affected by *Wnt11* RNAi.
(MP4)

Movie S4 **Time-lapse of DN-** ***Wnt11*** **RCAS electroporated embryo.** 175 μm thick slice of a HH20 embryo electroporated with DN-*Wnt11* RCAS construct (24 hours after transfection). Consistent with the *Wnt11* RNAi result and contrary to the control *EGFP* electroporation, very few migratory cells towards the subectodermal space and above the neural tube were observed. The migration of the EGFP –positive cells to the myotome is not affected by DN *Wnt11* plasmid.
(MP4)
