# Correction: Wnt11 Is Required for Oriented Migration of Dermogenic Progenitor Cells from the Dorsomedial Lip of the Avian Dermomyotome

**DOI:** 10.1371/journal.pone.0203913

**Published:** 2018-09-10

**Authors:** The PLOS ONE Staff

The figures and captions are in the incorrect order for Figs 1–8. Please see the corrected figures here. The publisher apologizes for these errors.

**Fig 1:**
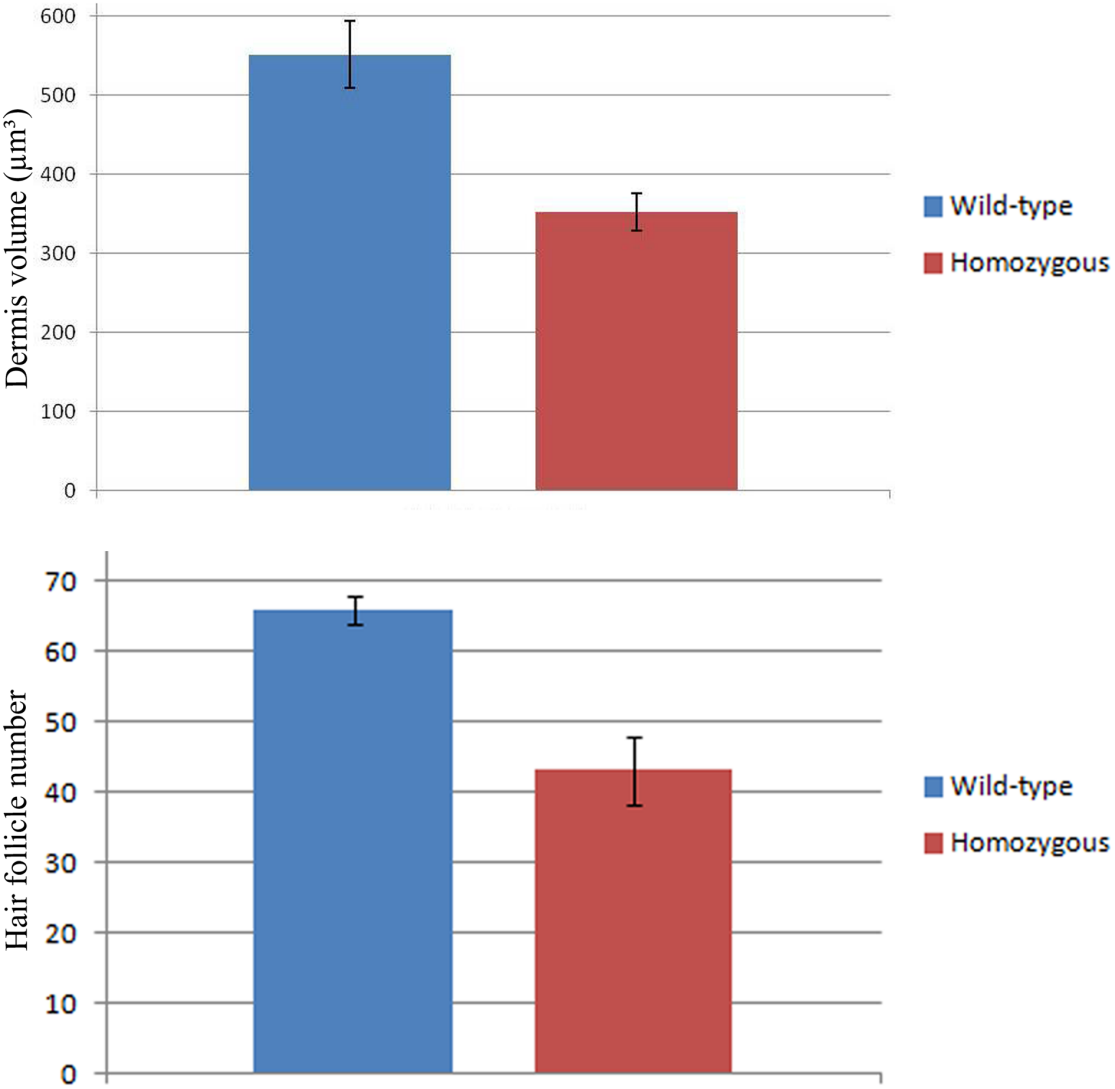
Murine dermis volume and hair follicles number comparison between *Wnt11* knock-out and wild-type mice. The diagram represents a graphical comparison of two averages with standard deviation bars of mice skin volume in sections of E18.5 mice wild-type and *Wnt11* knock-out mice. The results showed that the dermis volume in the sections of *Wnt11* knock-out mice was approximately 45% lesser when compared to the wild-type mice sections. The graphical comparison was performed in 20 sections after HE staining, 10 slides representing 3 mice for each genotype. The photos of the analysed sections were acquired at 40x magnification. For the graph representing the hair follicles number, the hair placodes on the back skin of the hybridized embryos for *mDermo-1* were counted in identical squares drawn on photos obtained using standardized magnification parameters. For this experiment, 6 *Wnt11* knock-out and 6 wild-type embryos were used. The diagram representing the graphical comparison of the two averages with standard deviation bars of hair follicles placodes number in mice embryos E14.5 hybridized for *mDermo-1*, shows a decrease of 35% in the number of hair follicles placodes in the mutant mice.

**Fig 2:**
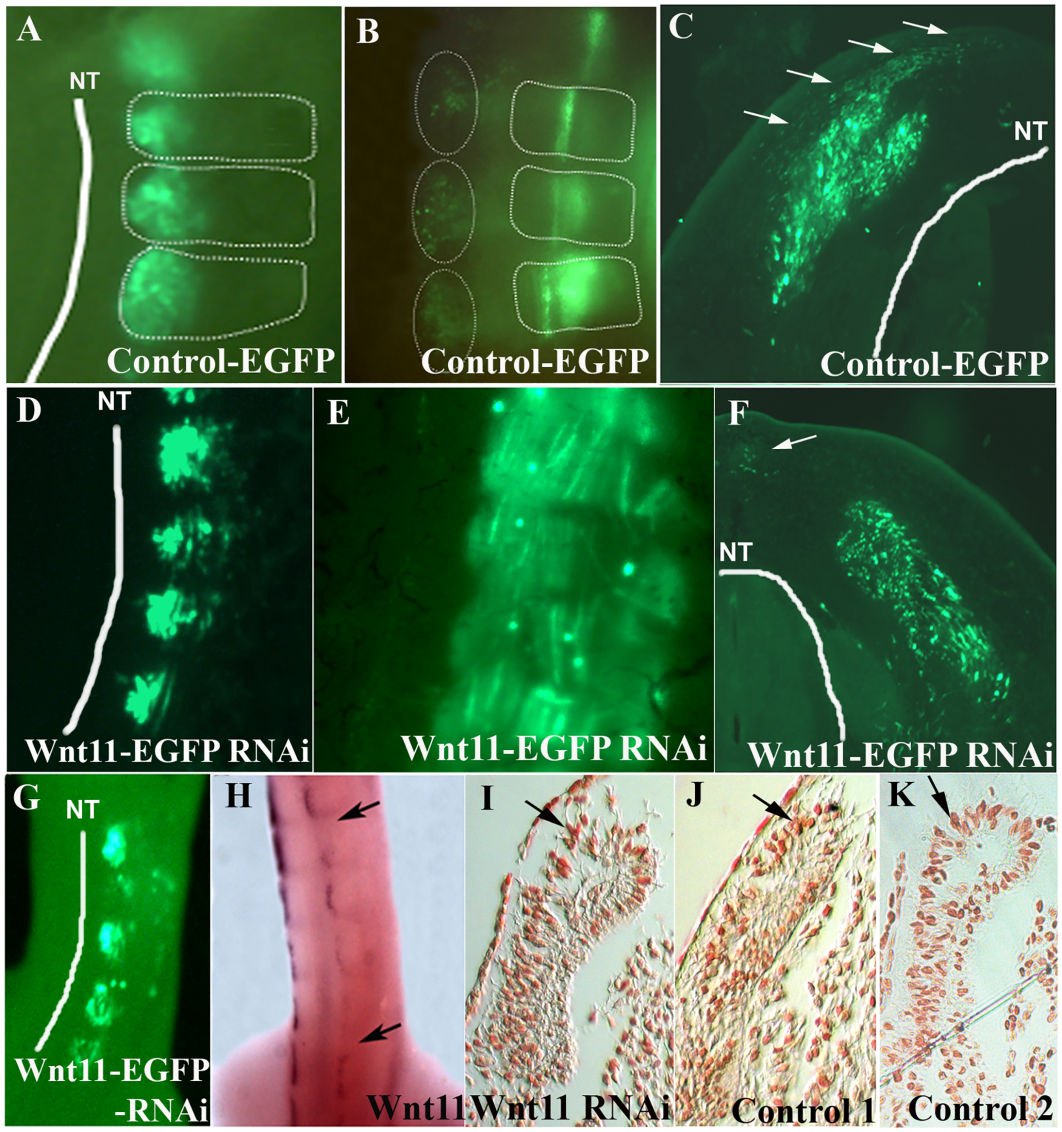
*Wnt11* expression in the DML is important for recruitment of dorsal dermal progenitors. A. EGFP-transfected DML of chicken embryo at stage HH18-19, 20 hours after electroporation. B. The embryo in A after 3 days of reincubation following electroporation. Note the presence of EGFP positive cells in the myotome and region of the future dorsal dermis (dotted circles). The white dotted lines squares delineate the somites. C. Cross-section of the embryo in B. EGFP-positive cells can be seen to be migrating into the subectodermal space overlying the spinal cord (white arrows). D. *Wnt11* RNAi on chicken embryo. DML of EFGP-*Wnt11* RNAi construct transfected chicken embryos at HH18-19, 20 hours after electroporation. E. After 3 days of reincubation following electroporation, the EGFP-*Wnt11* RNAi expressing cells are only to be found in the myotome in a disorganized manner, whereas EGFP-*Wnt11* RNAi positive cells are missing in the dorsal dermis anlage. F. In cross-section of the embryo in E, only very few cells (nearly undetectable) migrating from DML are present into the subectodermal space (white arrow) overlying the spinal cord (white line). White lines were traced along the neural tube for a better orientation. G. Targeting of the DML at stage HH14-17 by EGFP-*Wnt11* RNAi constructs after 24 hours reincubation and the corresponding *Wnt11* silencing as seen by ISH (area between black arrows in H). I. There is no evidence of increased cell death at the sites of EGFP-*Wnt11* construct transfection as seen by TUNEL staining. J. TUNEL staining of an electroporated embryo with scrambled DNA was used as control for our TUNEL assay. K. Represents the untreated control (Control 2) for TUNEL assay. Photos in I, J, K show the DML of the coressponding sections after TUNEL assay. Black arrows in I, J, K point towards Tunel-positive cells. NT: neural tube. DML: dorso-medial lip. Scale bar: 100 μm.

**Fig 3:**
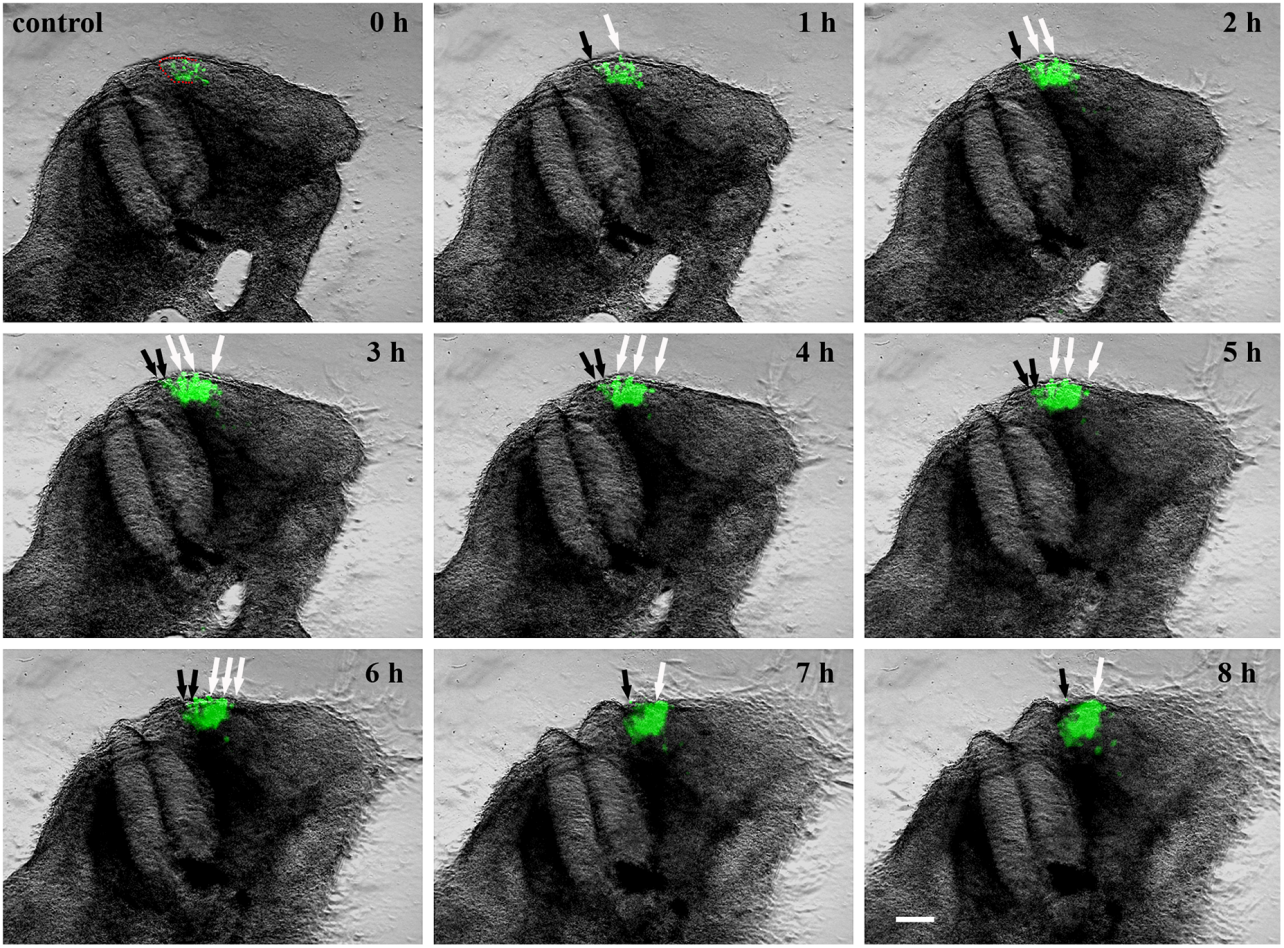
A series of 9 photos of a chicken embryo section electroporated with a control EGFP plasmid at the DML level and reincubated for 24 h. Each photo shows movement of cells in 1h time interval. At 0h the EGFP transfected cells are seen in the DML. At later time points, the dynamics of the transfected cells are visible, with cells moving to the subectodermal space above the neural tube (black arrows), and additionally migrating to the immediate neighbourhood of the neural tube (white arrows) and the myotome. These dynamic movements are characteristic of a normal distribution of DML cells, where DML cells migrate to the subectodermal space in order to form the dorsal dermis and to the myotome to form the muscle. The red outline indicates the DML. Scale bar: 100 μm.

**Fig 4:**
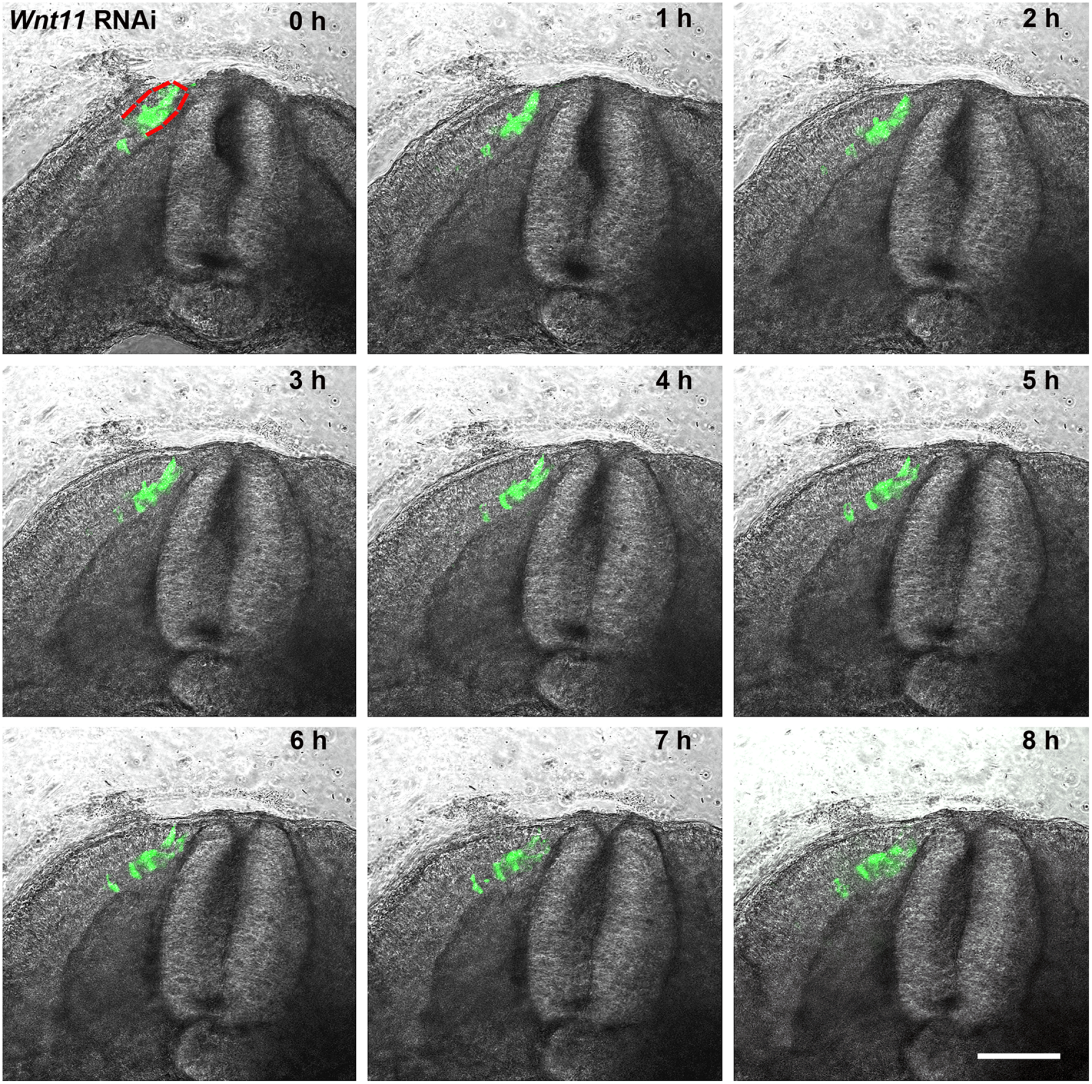
A series of 9 photos of a chicken embryo section electroporated with a *Wnt11* RNAi construct containing EGFP at the DML level, and then reincubated for 24h. Each photo shows movement of cells in 1h time interval. In contrast to the control experiment (Fig 2), the electroporated cells (green fluorescent) remain restricted to the DML or migrate into the myotome. No EGFP positive cells migrate towards the subectodermal space located above the neural tube or into the immediate neigbourhood. The absence of *Wnt11* in the DML thus results in a compromised EMT of the cells, which can only enter the myotome and no longer populate the subectodermal space above the neural tube. The red outline indicates the DML. Scale bar: 100 μm.

**Fig 5:**
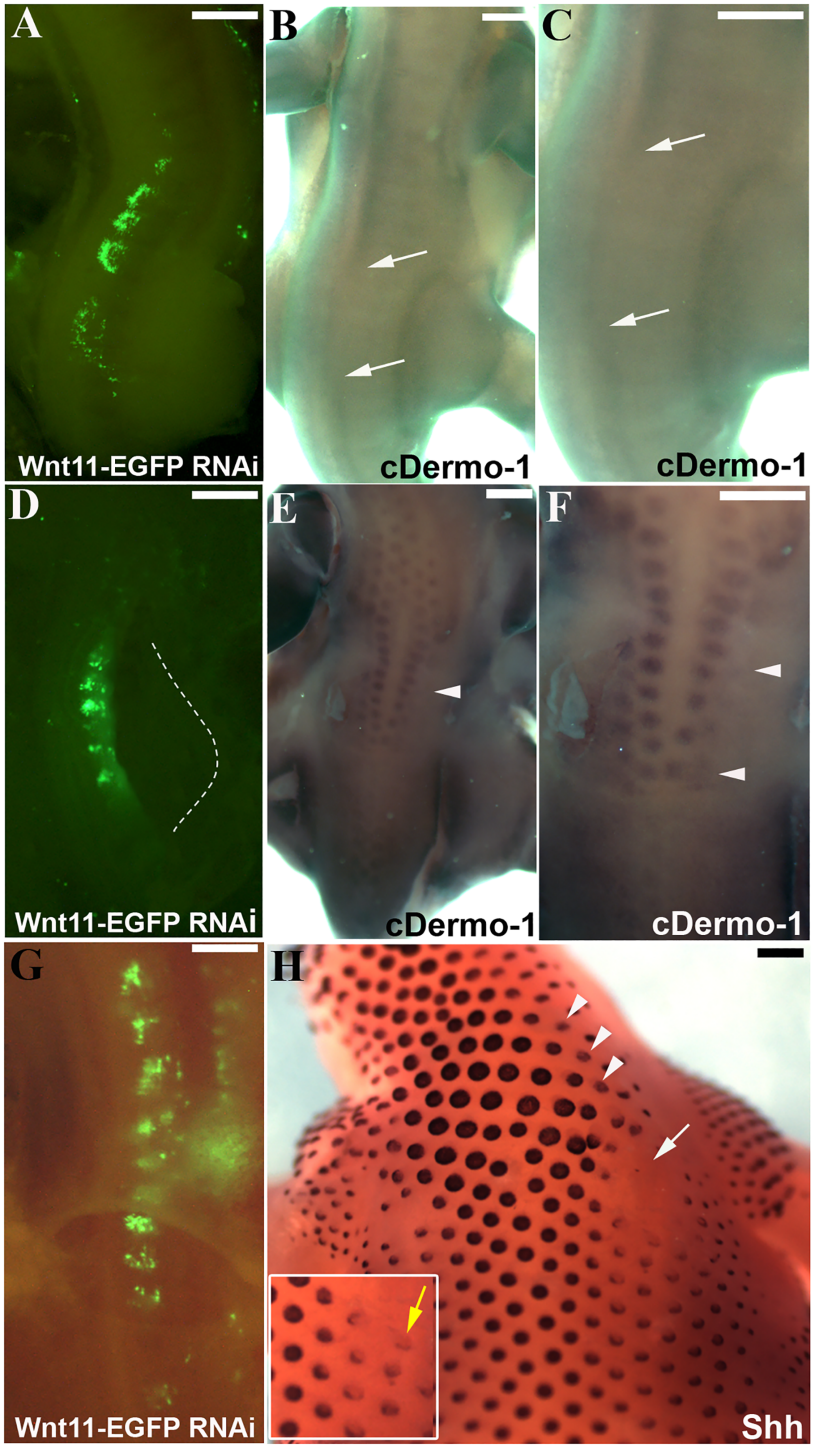
Decrease of dermal markers after *Wnt11* RNAi. A. Embryo showing the region of transfection after electroporation with EGFP-*Wnt11* RNAi. The embryo has been electroporated at stage HH14-17 and reincubated for 20 hours. B. The embryo in A after 2.5 days reincubation time following manipulation (HH28). Starting with stage 24, *cDermo-1* expression pattern can be found in the subectodermal mesenchyme of the trunk, and at stage 26 it is very strong along the dorsal midline. Following additional 2.5 days of reincubation after EGFP signal documentation for embryo in A, *cDermo-1* is remarkably reduced along the dorsal midline in the transfected region (area between white arrows in B). C. Higher magnification of the embryo in B. D. Electroporated embryo with *Wnt11* silencing construct at stage HH14-17 and photographed 20 hours later, at stage HH20. E. After longer reincubation periods following electroporation (4.5 days of the embryo in D), the embryo shows a retarded feather bud development as seen after *cDermo-1* ISH, which is expressed in this stage (HH31) in the mesenchyme of the nascent feather buds (the white arrowheads in E and F mark the missing row of feather buds on the manipulated side in D). F. Higher magnification of the embryo in E. G. Embryo showing the region of transfection after electroporation with EGFP-*Wnt11* RNAi at stage HH14-17 and photographed 24 hours later, at stage HH20. H. After 8 days reincubation for embryo in G after EGFP documentation (HH38) and hybridisation with a *Shh* probe we have noticed a retarded feather bud development as seen with cDermo-1. *In situ* hybridisation for *Shh* also reveals a changed morphology of the eventually formed feather buds, which are smaller and more flattened, with lower expression of *Shh* on the manipulated side (white arrow and arrowheads in H). The white square presents in a higher magnification the altered feather buds formation (yellow arrow). Scale bar: 100 μm.

**Fig 6:**
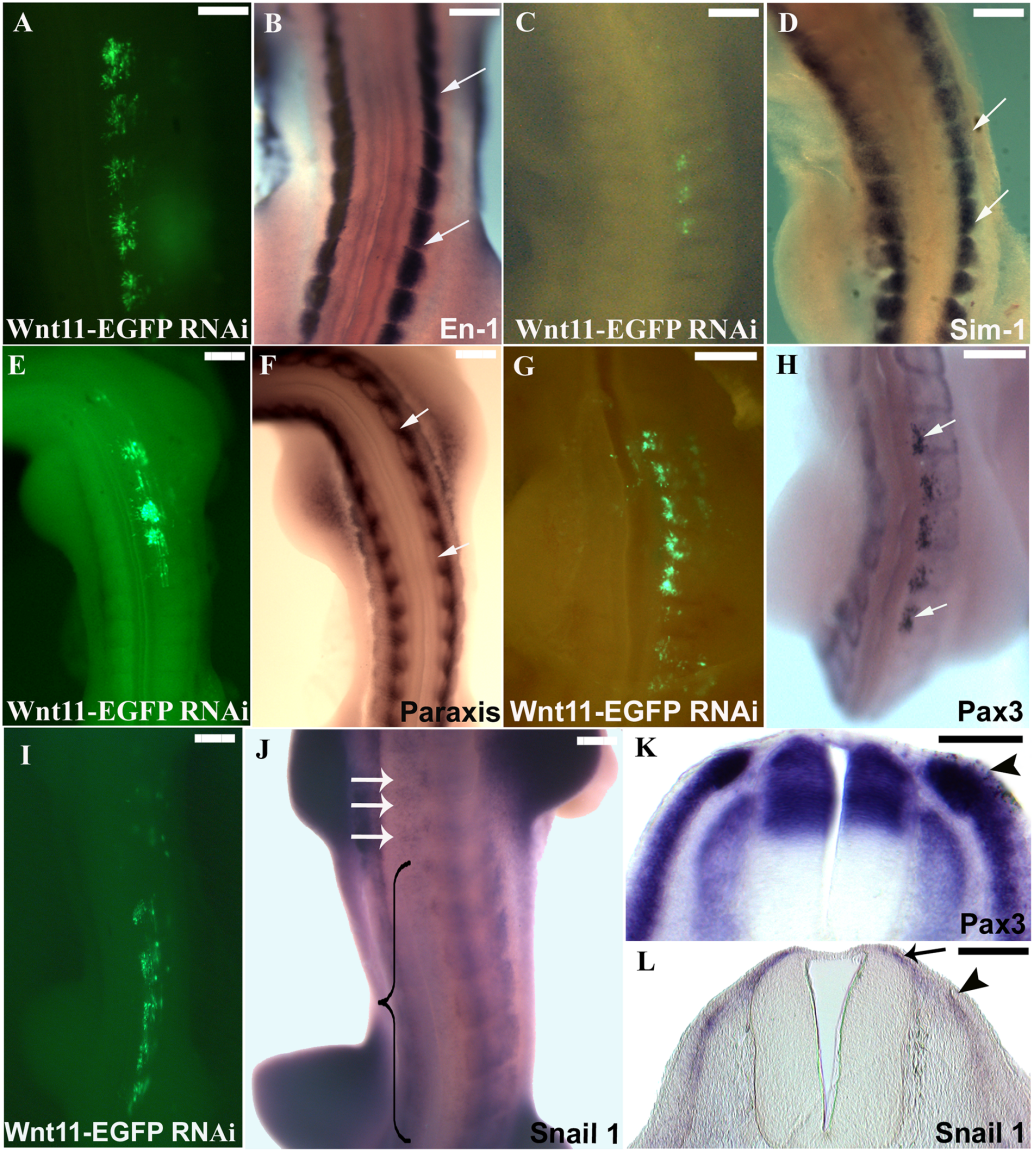
Effects of *Wnt11* silencing on dermomyotome. A, C, E, G and I. Chicken embryos electroporated with *Wnt11* RNAi at stage HH14-17 and after a reincubation period of 24 hours. B. The embryo in A (HH20) hybridized for *En-1*, shows that the central dermomyotomal compartment marked with *En-1* was unaffected in the electroporated area (the space between white arrows in B). D. The photo represents the embryo in C after ISH for *Sim-1* probe. The lateral dermomyotomal compartment marked with *Sim-1* (white arrows in D indicate the electroporated area) did not show any change in its expression after *Wnt11* silencing. F. ISH for *Paraxis* of the embryo presented in photo E. *Paraxis* transcripts seems not to be altered following *Wnt11* silencing in the DML (area between white arrows in F). H. The dermomyotomal marker Pax3, in contrast, is significantly upregulated at the site of *Wnt11* RNAi transfection (area between white arrows in H). K. The cross-section through the embryo in H in the manipulated area shows a strong upregulation of *Pax3* in the DML, while the DM remains normal when compared to the control side. I. Electroporated embryo at stage HH14-17 with *Wnt11* RNAi and after 24 hours reincubation (HH19). J. Hybridized embryo from photo I for *Snail1* probe. At stage HH20 the EMT has already started, and the *Snail1* expression can be seen in the myotome, dermomyotome, sclerotome and in the space above the neural tube (dermal progenitor cells). Whole-mount ISH of the embryo electroporated with *Wnt11* RNAi shows a decreased *Snail1* expression (the region in the bracket), while the white arrows point towards the normal expression of *Snail1* above the neural tube (dermogenic progenitors) at untreated levels. L. Section through the embryo in J in the affected region shows a decreased *Snail1* expression in the DML (black arrowhead) and above the neural tube (black arrow). Scale bar: 100 μm.

**Fig 7:**
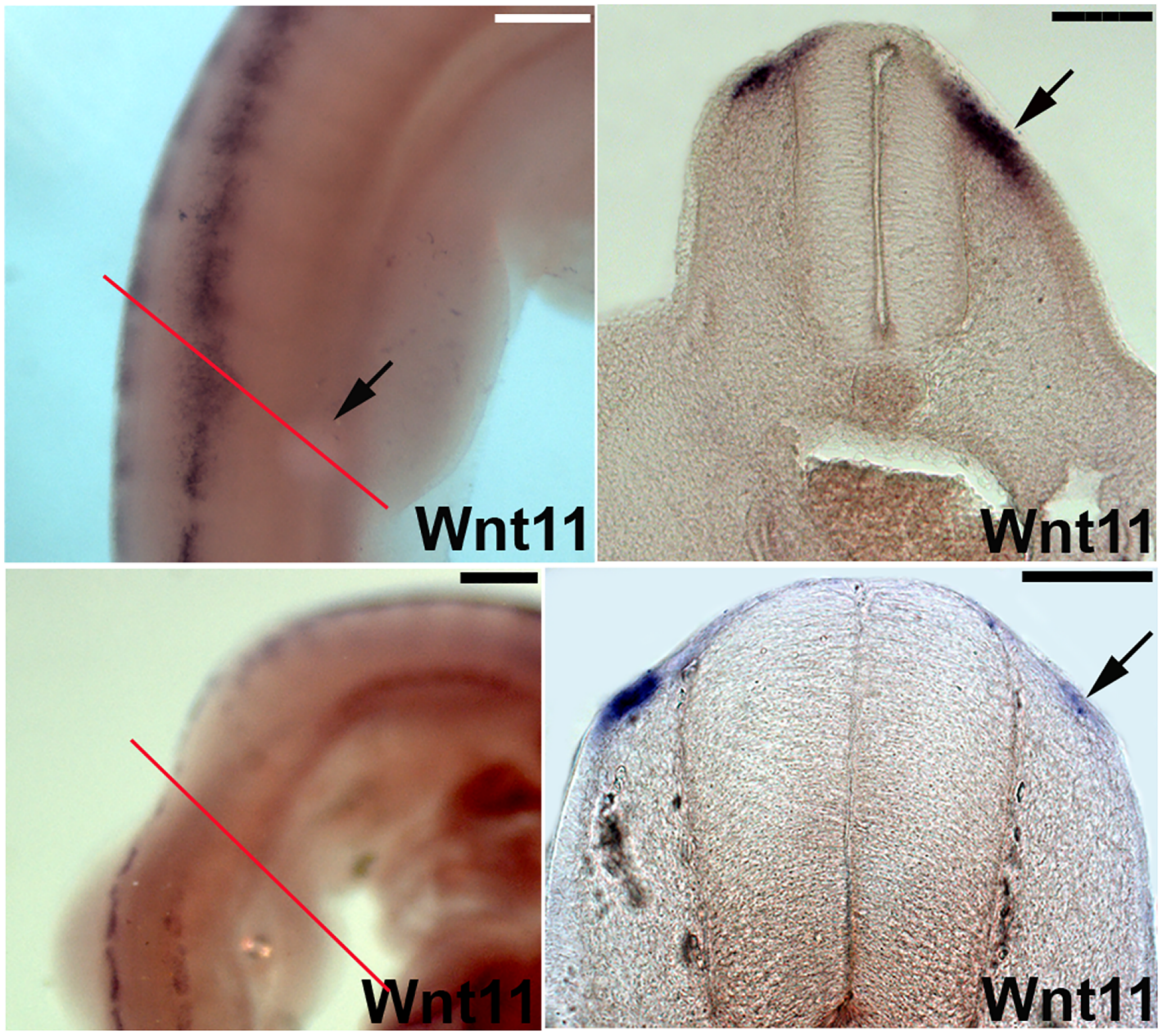
BMP2 signaling controls *Wnt11* expression in DML. A, B. BMP2 beads implanted into somites of chicken embryos (black arrow in A) lead to an upregulation of *Wnt11* as seen by ISH in vibratome sections (black arrow in B; section level indicated by red line in A). C, D. In contrast, grafting of Noggin cells (BMP antagonist) lead to a downregulation of *Wnt11* transcripts on the operated side (C) also visible in vibratome sections of the same embryo (black arrow in D; section level indicated by red line in C). Scale bar: 100 μm.

**Fig 8:**
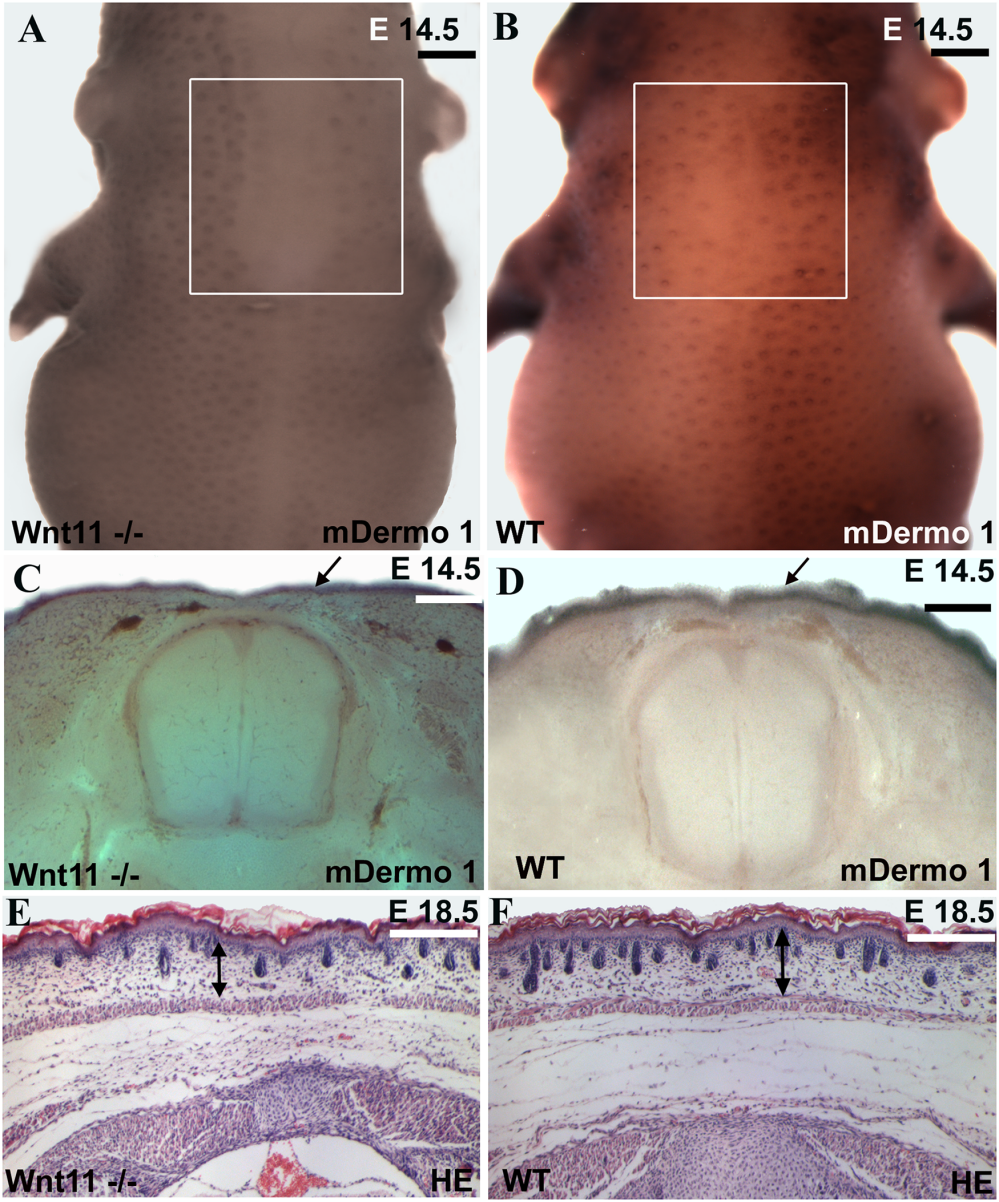
Dorsal dermis is decreased in dermis thickness and number of hair follicles in the Wnt11 knock-out mice. A, C, E. Homozygous *Wnt11* knock-out embryos. B, D, F. Wild-type embryos. Embryo in A represents a homozygous *Wnt11* knock-out embryo of embryonic day E 14.5. In the dorsal mid-line region of this embryo a large area devoid of hair placodes is detectable. The white square shows a decrease number of hair placodes in comparison to the WT embryo of similar developmental stage (B). Section through the embryo in A shows that the *cDermo-1* signal is diminished with corresponding decrease in dermal thickness in the mutant (black arrow in C) as compared with a similar section of the same magnification of a WT embryo (black arrow in D). Hematoxylin-Eosin staining in paraffin sections of knock-out mice of E 18.5 shows a remarkable decrease in dermis thickness (45%), as well as a reduced number of hair follicles (35%). (E) In comparison with a Hematoxylin-Eosin treated section of a WT mouse of the same developmental stage (F). Double-headed arrows in E and F show the remarkable difference in dermis thickness between the mutant mouse embryo as compared to the normal thickness of dermis in a wild-type embryo (photographs were taken at the same magnification). The sections were taken at the trunk regions of the mice. The analysis was restricted to the same anatomical area of the dorsal skin for all embryos investigated. Scale bar: 100 μm.
