# Supplementary figures and images for: Wnt11 Is Required for Oriented Migration of Dermogenic Progenitor Cells from the Dorsomedial Lip of the Avian Dermomyotome
Source: PLoS One. 2014 Mar 26;9(3):e92679. doi: 10.1371/journal.pone.0092679 (PMC3966816; doi:10.1371/journal.pone.0092679)

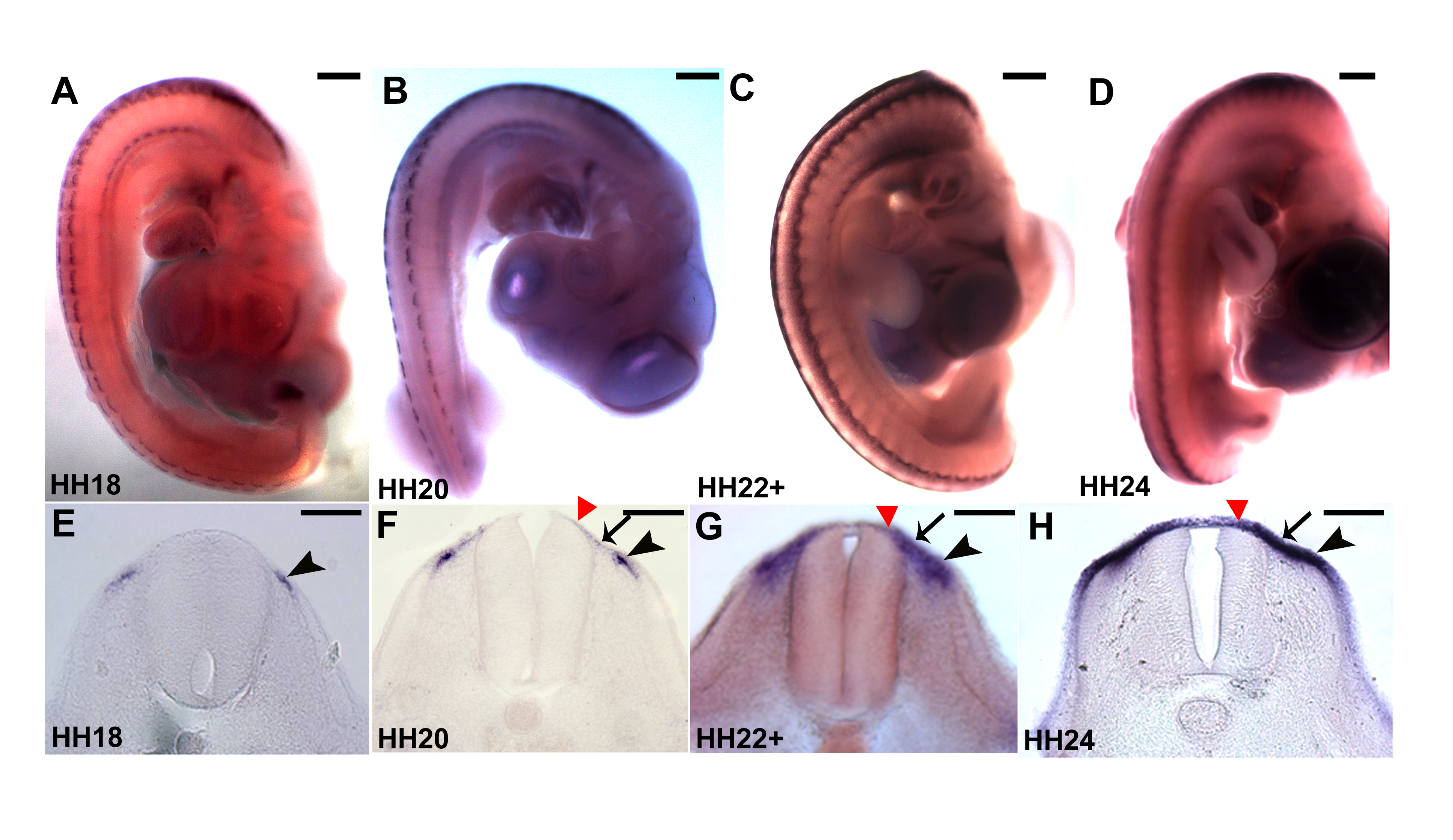

Supplement: Figure S1 — Expression of Wnt11. A. Whole-mount ISH for Wnt11 at stage HH18. E. Cross-section through the embryo in A. Expression of Wnt11 is limited to the dorso-medial lip of the dermomyotome (black arrowhead). C. Whole-mount ISH for Wnt11 at stage HH20. As the embryo develops, the expression domain extends more caudally, retaining its prominent expression cranially. F. In the cross-section of the embryo in B, we can observe a stronger expression in the dorso-medial lip (black arrowhead), some cells Wnt11 positive are located between the dorso-medial lip and the neural tube (black arrow) and on top of the neural tube (red arrowhead). C. The whole-mount ISH for an HH22+ stage embryo shows a prominent expression of the Wnt11 transcripts, maintaining its cranio-caudal gradient. G. The cross-section through the embryo HH22+ reveals a broader area of the Wnt11 gene expression, including the area between the DML and neural tube, the anlage of the future dorsal dermis (black arrow) and above the neural tube (red arrowhead). Its expression pattern in the dorso-medial lip is also increased. D. Whole-mount expression of a stage HH24 embryo. The strong expression of the Wnt11 transcripts becomes obvious. H. The cross-section of the HH24 stage embryo hybridised for the Wnt11 gene shows clearly the large area of cells positive for Wnt11, including the subectodermal space (black arrow and black arrowhead) and also an increased expression on top of the neural tube (red arrowhead), which later on will form the dorsal dermis. All the sections showed in this figure were performed at the interlimb level. Scale bar: 100 μm. (TIF) [file pone.0092679.s001.tif]
