# Supplementary material for: Wnt11 Is Required for Oriented Migration of Dermogenic Progenitor Cells from the Dorsomedial Lip of the Avian Dermomyotome
Source: PLoS One. 2014 Mar 26;9(3):e92679. doi: 10.1371/journal.pone.0092679 (PMC3966816; doi:10.1371/journal.pone.0092679)
Supplement: Table S1 — Selected nucleotide sequences for silencing of Wnt11 mRNA. This table presents the four selected target sites. The first target site starts at +217 nt, the second target site starts at +326 nt, the third target site starts at +761 nt and the fourth target site starts at +1034 nt. The forward and reverse strands of the shRNA inserts were designed as presented in this table. (PDF) [file pone.0092679.s002.pdf]

Table S1

Target sequences for *Wnt11* RNAi:

Target 1:

Forward_Wnt11-217

5'GATCCGCACGGGAAGTCATAAAGATTCAAGAGATCTTTATGACTTCCCGTGCTTTTTTGAATTCA 3'

Reverse_Wnt11-217

5'AGCTTGAATTCAAAAAAGCACGGGAAGTCATAAAGATCTCTTGAATCTTTATGACTTCCCGTGCG 3'

Target 2:

Forward_Wnt11-326

5'GATCCGGAGTCAGCATTTGTGTATTTCAAGAGAATACACAAATGCTGACTCCTTTTTTGAATTCA 3'

Reverse_Wnt11-326

5'AGCTTGAATTCAAAAAAGGAGTCAGCATTTGTGTATTCTCTTGAAATACACAAATGCTGACTCCG 3'

Target 3:

Forward_Wnt11-761

5'GATCCGCAAATACCTCGTGCCAAATTCAAGAGATTTGGCACGAGGTATTTGCTTTTTTGAATTCA 3'

Reverse_Wnt11-761

5'AGCTTGAATTCAAAAAAGCAAATACCTCGTGCCAAATCTCTTGAATTTGGCACGAGGTATTTGCG 3'

Target 4:

Forward_Wnt11-1034

5'GATCCAGAGGACTGTCGAGAGATATTCAAGAGATATCTCTCGACAGTCCTCTTTTTTTGAATTCA 3'

Reverse_Wnt11-1034

5'AGCTTGAATTCAAAAAAAGAGGACTGTCGAGAGATATCTCTTGAATATCTCTCGACAGTCCTCTG 3'
