# Supplementary material for: Wnt11 Is Required for Oriented Migration of Dermogenic Progenitor Cells from the Dorsomedial Lip of the Avian Dermomyotome
Source: PLoS One. 2014 Mar 26;9(3):e92679. doi: 10.1371/journal.pone.0092679 (PMC3966816; doi:10.1371/journal.pone.0092679)
Supplement: Table S2 — Primers used for qPCR. Specific primer pairs used for qPCR of the genes analysed. 18sRNA was used as a reference gene. (PDF) [file pone.0092679.s003.pdf]

Table S2

Real -Time PCR

Total RNA was extracted and reverse transcribed. Upstream and reverse primers are summarized here.

Gene	Primer sense [5‘-3‘]	Primer antisense [5‘-3‘]	amplicon size [bp]	Accession Number
18s rRNA	catggtgaccacgggtgac	ttccttggatgtggtagccg	82	53990
GAPDH	cagcctcgtcccgtagaca	cgctcctggaagatggtgat	234	126012538
ß-actin	gccaacacagtgctgtctggtggtac	cacacagagtacttgcgctcaggagg	137	145966868
actg1	accaacagcagacttccaggat	agactggcaagaaggagtggtaa	79	48762677
ubc	aggtcaaacaggaagacagacgta	tcacacccaagaacaagcaca	80	149254583
wnt11	ctgaatcagacgcaacactgtaaac	ctctctccaggtcaagcaggtag	205 bp	NM_009519.2
twist1	agctacgccttctccgtct	tccttctctggaaacaatgaca	123 bp	NM_011658.2
twist2	catgtccgcctcccacta	ggtgccgaaagtcacagc	123 bp	NM_007855.2
Fgf10	cgggaccaagaatgaagact	gcaacaactccgatttccac	69 bp	NM_008002.4
Krt5	cagagctgaggaacatgcag	cattctcagccgtggtacg	512 bp	NM_027011.2
